# Decoding *Zmiz1*: a key transcription coactivator in development and disease

**DOI:** 10.3389/fcell.2026.1933470

**Published:** 2026-10-08

**Authors:** Rajan K C, Anoushka Shenoy, Most Ayesha Siddika, Nehal R. Patel, Stryder M. Meadows, Maria J. Galazo

**Affiliations:** 1 Department of Cell and Molecular Biology, Tulane University, New Orleans, LA, United States; 2 Tulane Brain Institute, Tulane University, New Orleans, LA, United States

**Keywords:** autism spectrum disorder, neurodevelopmental disorders, transcriptional regulation, vascular development, Zmiz1

## Abstract

ZMIZ1, a zinc finger MIZ-type transcriptional co-activator in the PIAS family, is emerging as a central regulator at the interface of development and disease. Through interactions with key signaling effectors, including NOTCH1, SMAD3/4, p53, estrogen receptor, and androgen receptor, ZMIZ1 shapes transcriptional programs that govern embryogenesis, vascular and lymphatic development, and cerebral cortex development. Disruption of these programs is increasingly linked to human pathology: pathogenic ZMIZ1 variants cause a syndromic neurodevelopmental disorder with autism spectrum disorder and intellectual disability, and are associated with Hirschsprung disease, depression, and Lewy body disease. Beyond the nervous system, genetic and functional studies implicate ZMIZ1 in T-cell development and leukemia, multiple autoimmune diseases, type-2 diabetes, and a broad spectrum of cancers, including cutaneous squamous cell carcinoma, prostate, breast, colorectal cancer, osteosarcoma, and glioblastoma. Despite this expanding disease spectrum, the cell type-specific and context-dependent mechanisms by which ZMIZ1 integrates activation state from different signaling pathways, chromatin regulation, and transcription activation remain poorly understood. In this review, we synthesize current knowledge of ZMIZ1 and molecular regulatory networks across developmental, neurological, immune, metabolic, and oncologic contexts. We highlight convergent themes in ZMIZ1 biology, outstanding mechanistic questions, and emerging evidence supporting ZMIZ1 and its downstream pathways as potential diagnostic, prognostic, and therapeutic targets in human disease. By integrating genetic, molecular, and systems-level evidence across diverse organs and diseases, this review proposes a working model of ZMIZ1 as a partner-directed, dosage-sensitive transcriptional rheostat, identifies experiments required to test it, and appraises the current, largely preclinical, evidence for its clinical potential.

## 1 Introduction

The precise control of gene expression is fundamental to how cells acquire their identities, respond to environmental cues, and maintain tissue homeostasis. While decades of research unfolded the transcriptional mechanisms, the field now faces a pivotal challenge: deciphering how co-regulatory proteins integrate multimodal inputs to instruct specific developmental trajectories while avoiding pathological derailments (Talukdar and Chatterji, 2023; Stallcup and Poulard, et al., 2020). This intricate balance is essential for maintaining cellular homeostasis, and disruptions can result in a wide array of diseases. Members of the PIAS (protein inhibitors of activated STAT) family translate inputs from Notch, TGF-β/SMAD, p53, nuclear hormone receptors, and other signaling pathways into coordinated transcriptional programs that shape embryonic patterning, as well as vascular, immune, and nervous system development. Perturbations in these molecular integrators and regulatory hubs can therefore produce pleiotropic phenotypes that span neurodevelopmental disorders, autoimmunity, metabolic disease, and cancer.

Zinc finger MIZ-type containing one is a PIAS-related transcriptional co-activator that is emerging as a central node in this regulatory landscape. Structurally, ZMIZ1 contains an N-terminal tetratricopeptide repeat (TPR) region, a central MIZ/SP-RING zinc-binding domain, and a C-terminal transactivation domain, enabling it to scaffold protein–protein interactions and modulate chromatin accessibility. Biochemically, ZMIZ1 has been shown to potentiate the activity of several major transcription factors, including NOTCH1 (Pinnell et al., 2015; Wang et al., 2018), SMAD3/4 (Li et al., 2006), p53 (Lee et al., 2007), the estrogen receptor (Zhao et al., 2024), and the androgen receptor (Li et al., 2011; Sharma et al., 2003), positioning it at the intersection of developmental and stress-response pathways. Genetic and functional studies now demonstrate that ZMIZ1 regulates diverse biological processes, such as embryogenesis, lymphatic and blood vascular development, neurogenesis and synaptic connectivity, hematopoiesis, and T-cell maturation, and that it can act as both a transcriptional co-activator and chromatin remodeler in a context-dependent manner (Pinnell et al., 2015; Wang et al., 2018; Beliakoff et al., 2008; Rodríguez-Magadán et al., 2010; Carapito et al., 2019; K C et al., 2024b).

Converging human genetic, genomic, and experimental data highlight the clinical importance of this multifunctional regulator. *De novo* and inherited pathogenic variants in ZMIZ1 cause a syndromic neurodevelopmental disorder characterized by intellectual disability, autism spectrum disorder with additional congenital anomalies (Carapito et al., 2019; Valind et al., 2021; Latchman et al., 2020; Córdova-Fletes et al., 2015; Phetthong et al., 2021; Cormier et al., 2025), and ZMIZ1 has been implicated in Hirschsprung disease, depression, and Lewy body disease (Valind et al., 2021; Wei et al., 2021; Hou et al., 2023). Genome-wide association studies further link common ZMIZ1 variation to multiple sclerosis, vitiligo, inflammatory bowel disease, and type 2 diabetes (Fewings et al., 2017; Lu et al., 2024; Sun et al., 2014; Jakobsen et al., 2014; Dubois et al., 2010; Van et al., 2015; Andersen et al., 2014; the DIAbetes Genetics Replication And Meta-analysis DIAGRAM Consortium et al., 2012), while somatic alterations and dysregulated expression of ZMIZ1 have been described in a broad range of malignancies, including T-cell acute lymphoblastic leukemia, tongue and cutaneous squamous cell carcinoma, and hormone-driven cancers such as breast and prostate cancer (Pinnell et al., 2015; Shan et al., 2019; Zhang et al., 2022a; Aiderus et al., 2021; Genetics and Epidemiology of Colorectal Cancer Consortium GECCO et al., 2014; Zhou Y. et al., 2022; Mathios et al., 2019).

Despite this expanding disease spectrum, we still lack a unified framework that explains how ZMIZ1 integrates upstream signals, interacts with chromatin machinery, and controls cell type-specific transcriptional networks across organs and life stages. In this review, we propose such a framework as an explicit working model. We discuss ZMIZ1’s roles in embryogenesis, nervous system development, vascular and immune regulation, metabolism, and cancer, and highlight convergent mechanistic themes, outstanding questions, and opportunities to target *Zmiz1*-regulated pathways for diagnosis and therapy. By integrating developmental biology, neurobiology, cancer biology, and clinical genetics, this work establishes ZMIZ1 as a pivotal gene for understanding transcriptional coordination in health and disease, charting a course for mechanism-driven research and potential therapeutic development for neurodevelopmental disorders.

### 1.1 A unifying framework: ZMIZ1 as a partner-directed transcriptional rheostat

A recurring difficulty in interpreting the ZMIZ1 literature is that the protein appears linked to a remarkably broad and seemingly unrelated set of diseases. We propose a conceptual framework in which these diverse observations can be integrated under a common organizing principle: ZMIZ1 functions as a partner-directed transcriptional rheostat. ZMIZ1 lacks intrinsic sequence-specific DNA-binding activity and instead acts by being recruited to chromatin by partner transcription factors–including NOTCH1, SMAD3/4, p53, the estrogen and androgen receptors, PROX1, and the PIAS–MITF axis–where it amplifies or fine-tunes the transcriptional program those partners direct. Consequently, the biological output of ZMIZ1 is not fixed, but could be set by two variables: which partner is active in a given cell type (context- and cell-type-dependence), and the dosage of functional ZMIZ1 available (dosage-dependence).

These two axes organize the remainder of this review. The partner-dependence axis explains how the same co-activator can specify lymphatic identity through PROX1 in endothelial cells, drive Notch-dependent MYC induction in T-cell progenitors, and promote estrogen-driven proliferation in breast epithelium. Therefore, the partner-dependence axis is linked to ZMIZ1’s variable responses across cell types. The dosage axis reconciles the apparent paradox between loss- and gain-of-function phenotypes: heterozygous loss or haploinsufficiency of ZMIZ1 produces a syndromic neurodevelopmental disorder, whereas over-expression or expression of an N-terminally truncated stabilized isoform drives cutaneous carcinomas. Both loss- and gain-of-function lead to pathological phenotypes, strongly suggesting that ZMIZ1 dosage operates within strict margins, specific for each partner and cellular context (Figure 1).

**FIGURE 1:**
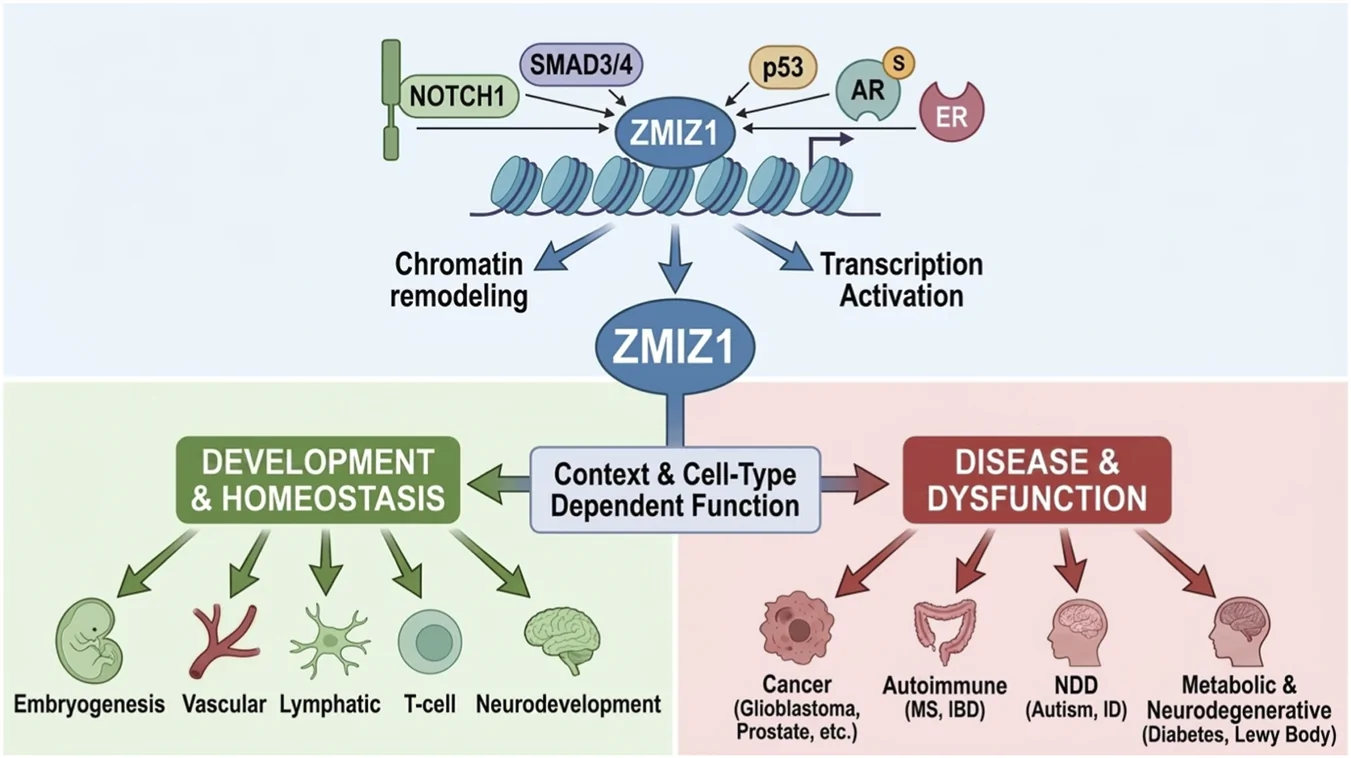
Unifying model of ZMIZ1 as a context- and cell-type-dependent transcriptional co-activator bridging normal development and disease. This figure presents the central framework of the review: how a single modular co-regulator integrates distinct upstream signals at chromatin and, depending on cellular context, drives either physiological programs or pathological outcomes. At chromatin (nucleosome array), ZMIZ1 acts as a signal-responsive co-activator that potentiates NOTCH1, the TGF-β effectors SMAD3/4, the tumor suppressor p53, the androgen receptor (AR; shown with its SUMO modification, “S”), and the estrogen receptor (ER), thereby promoting chromatin remodeling and transcriptional activation of target-gene programs. The convergent output is a context- and cell-type-dependent function: the organizing principle that reconciles ZMIZ1’s apparently disparate roles: the same regulatory machinery is deployed to different ends according to tissue, developmental stage, and partner availability. In normal contexts (green branch), ZMIZ1 supports embryogenesis, blood-vascular and lymphatic development, T-cell development, and neurodevelopment. When ZMIZ1 or its downstream programs are dysregulated (red branch), the same capacity contributes to cancer (e.g., glioblastoma, prostate), autoimmune disease (multiple sclerosis, inflammatory bowel disease), neurodevelopmental disorders (autism spectrum disorder, intellectual disability), and metabolic/neurodegenerative disease (type 2 diabetes, Lewy body disease). The model emphasizes that developmental and pathological outcomes are two faces of the same context-gated molecular logic, and that both established (NOTCH1, nuclear receptors) and incompletely defined pathways contribute to disease, highlighting priorities for therapeutic intervention. Created with BioRender.com.

Throughout the sections that follow, we indicate the potential operative partners and the direction of the ZMIZ1 dosage change for each context, and we return to this framework when discussing convergent themes and the opposing roles of ZMIZ1. We state the nature of the evidence supporting ZMIZ1’s proposed functions, interacting partners, and associations with disorders, and we suggest potential mechanisms linking the proposed ZMIZ1 transcriptional rheostat framework to phenotypes in different pathological contexts.

To keep mechanistic conclusions distinct from statistical correlations, we also grade the supporting data into three tiers: Tier 1 (causal), such as evidence from pathogenic human variants and experimental functional evidence from conditional animal models with gain- and/or loss-of-function; Tier 2 (functional associative) such as expression changes linked to phenotype in models or patient tissue; and Tier 3 (genetic association only), in case of GWAS or eQTL loci that mark, but do not yet prove a causal role for ZMIZ1 (Table 1). Only a limited number of studies have comprehensively examined ZMIZ1-interacting partners, changes in ZMIZ1 dosage or expression, downstream effectors, and resulting phenotypes within the same experimental context. Therefore, the conceptual framework proposed here, which integrates evidence from diverse studies, should be viewed as a working model rather than an established mechanism. This framework provides a basis for interpreting the diverse functions of ZMIZ1 and generating testable hypotheses, and can be refined as additional evidence becomes available.

**TABLE 1:** **Main ZMIZ1–disease relationships graded by evidence tier and direction of effect.** Tier 1, causal (pathogenic human variants and experimental functional evidence from conditional animal models with gain- and/or loss-of-function); Tier 2, functional or associative (expression changes linked to phenotype); Tier 3, genetic association only (GWAS/eQTL loci). Multiple tiers indicate that multiple studies, with different degrees of evidence, support the observations. The column “ZMIZ1 change” indicates changes in ZMIZ1 expression and, if known, how the change drives the phenotype. Abbreviations: ASD, autism spectrum disorder; EC, endothelial cell; HDLEC, human dermal lymphatic endothelial cell; ID, intellectual disability; KD, knockdown; KO, knockout; LEC, lymphatic endothelial cell; NDD, neurodevelopmental disorder; SCC, squamous cell carcinoma; T-ALL, T-cell acute lymphoblastic leukemia; TK, tyrosine kinase; TKI, tyrosine kinase inhibitor.

Condition/phenotype	System/model	Evidence type	Tier	ZMIZ1 change (direction)	Proposed partner/mechanism
Syndromic NDD (ID, ASD)	Human *de novo* variants; mouse cortex KO	Causal variants + conditional KO with rescue	1	Loss/haploinsufficiency = cause phenotype	Chromatin and transcription; Efnb2, Auts2, Lhx2 targets
Blood-vascular development	Mouse and zebrafish KO	Global/EC-specific KO	1	Loss = cause phenotype	GATA2/3, Fra-1, angiogenic gene program
Lymphatic development	Mouse LEC KO; human HDLEC	Conditional KO + chromatin accessibility	1	Loss = cause phenotype	PROX1 promoter/enhancer regulation
T-cell development/T-ALL	Mouse models; human leukemia	Mechanistic + functional	1 and 2	Gain (oncogenic)	NOTCH1 cofactor – MYC induction (TPR domain)
Leukemia (ZMIZ1-ABL1 fusion)	Human case reports	Case reports	3	Fusion protein/gain of TK activity = higher risk	ABL1 tyrosine kinase (TKI-sensitive)
Type 2 diabetes/islet function	Human GWAS/eQTL; mouse β-cell KO	Association + functional	2 and 3	Locus assoc.; loss and gain impair secretion	Insulin exocytosis; islet differentiation gene expression
Multiple sclerosis	Human GWAS/expression	Genetic association	3	Low expression = higher risk	Vitamin D response; immune-gene network
Vitiligo	Human GWAS; melanocyte lines	Association + functional	2 and 3	Loss or KD = higher risk	PIAS3–MITF axis; mtOR/akt/gsk-3β
Inflammatory bowel disease	Human GWAS/eQTL	Genetic association	3	Mixed direction	T-cell-specific eQTL
Cutaneous SCC	Mouse transposon screen; human tumors	Functional (driver)	2	Gain/truncation (Zmiz1Δ185) = higher risk	Cooperates with chromatin-remodeler loss
Prostate cancer	Human GWAS/methylation; cell lines	Association + functional	2 and 3	Gain (incl. CircZMIZ1 and potentiation of AR) = higher risk	Androgen receptor coactivation (short polyQ)
Breast cancer (ER+)	Human tumors; cell lines	Functional	2	Gain = higher risk	Estrogen receptor (ERα) coactivation; E2F2
Colorectal cancer	Human GWAS; cell lines	Association + functional	2 and 3	Reduced in tumor tissue; KD anti-proliferative	SIRT1/FOXO3a axis; *ZMIZ1*-AS1 locus
Glioblastoma	Human tumors; *in vitro*/*in vivo*	Functional	2	Silencing hyperstable isoform (hypermethylation) = better survival. KD reduces migration	Intragenic DNA methylation
Lewy body disease	Human GWAS	Genetic association	3	Variant protective	Mitophagy (p-S65-Ub); APOE4 interaction

### 1.2 ZMIZ1: general overview

ZMIZ1 (also known as ZIMP10, MIZ, RAI17, or KIAA1224) is a transcriptional co-activator protein that shapes gene expression programs across multiple tissues and developmental stages. *ZMIZ1* coding sequence was first identified in 1999 as KIAA1224 (Nagase et al., 2000). This gene codes for a non-canonical member of the PIAS family of proteins. The PIAS family contains seven members, including ZMIZ1, ZMIZ2, PIAS1, PIAS2, PIAS3, PIAS4, and NSMCE2.

ZMIZ1 belongs to the Zinc finger MIZ-type family of proteins since it contains a SP-RING zinc finger or MIZ domain, which confers SUMO-E3-ligase activity and is highly conserved in the PIAS family. ZMIZ1 similarity with canonical members of the PIAS family is limited to this domain. Structurally and functionally, ZMIZ1 differs substantially from canonical PIAS (protein inhibitor of STAT) proteins, which function as co-regulators of many transcription factors, enhancing or repressing transcriptional output, mainly by SUMO protein modification. PIAS proteins covalently attach SUMO to lysine residues on their targets via SUMO-E3-ligase activity from their SP-RING domain (Rytinki et al., 2009). However, evidence for ZMIZ1 regulation via SUMOylation is less well established compared to the robust SUMO ligase activity of canonical PIAS (Lomelí, 2022). ZMIZ1 is widely considered a co-activator, acting mostly via direct binding and regulation of DNA-binding transcription factors. ZMIZ1 has a modular structure with multiple domains that enable its function as co-activator (Figure 2): 1) an N-terminal tetratricopeptide repeat (TPR) domain, unique to ZMIZ1 among the MIZ proteins. This protein-protein interaction domain is critical for its interaction with NOTCH1 (Pinnell et al., 2015; Lomelí, 2022). 2) the SP-RING-type zinc finger region, or MIZ domain, is important for protein–protein interactions and SUMOylation. ZMIZ1 can enhance SUMOylation of the androgen receptor and increase its transcriptional activity (Sharma et al., 2003). 3) Intrinsically disordered regions, which promote liquid-liquid phase separation and formation of multimolecular assemblies (Lomelí, 2022; K C et al., 2024a). 4) C-terminal proline-rich transactivation domain (TAD) that can activate multiple transcription factors, including the androgen receptor, SMAD3, and p53 (Lomelí, 2022; K C et al., 2025). Canonical PIAS proteins do not have a TAD (Rytinki et al., 2009). More details on ZMIZ1 and canonical PIAS protein structure and domains are described in (Lomelí, 2022, K C et al., 2024a).

**FIGURE 2:**
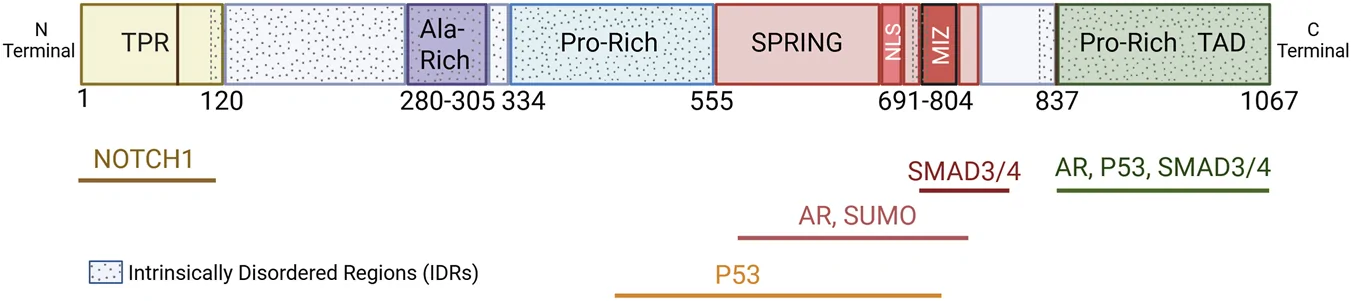
ZMIZ1 protein domains and interacting partners Schematic of ZMIZ1 and protein domains: N-terminal tetratricopeptide repeat (TPR), Alanine-rich domain (Ala-Rich), Proline-rich domain (Pro-Rich), SP-RING/MIZ type zinc finger domain (SPRING/MIZ), nuclear localization signal (NLS), transactivation domain (TAD). Dotted areas correspond to intrinsically disorder regions (IDRs). Numbers under ZMIZ1 denote amino acid residues at the beginning and end of each labeled domain. Below ZMIZ1, the colored lines and ZMIZ1’s interaction partners (NOTCH1, P53, AR, SUMO, SMAD3/4) align to the structural domain in ZMIZ1 known to mediate their interaction.

Given this structure-function logic, ZMIZ1 is well-positioned to serve as a context-dependent amplifier or modulator of major signaling-responsive transcription factors (Figure 1). This overview sets the stage for the next section examining how ZMIZ1’s regulatory capacity is deployed in embryogenesis, vascular and lymphatic biology, cerebral cortex development, immune lineages, metabolism, and cancer.

### 1.3 Nomenclature

Throughout this review, we refer to Zinc Finger MIZ-Type Containing one by its official gene symbol in the National Center for Biotechnology Information’s “Entrez Gene” database (https://www.ncbi.nlm.nih.gov/datasets/gene/57178) (ZMIZ1). We followed standard nomenclature rules for references to the human gene and protein: ZMIZ1 (gene), ZMIZ1 (protein); and for references to the mouse gene and protein: Zmiz1 (gene), ZMIZ1 (protein). For references to the protein in a general context that applies to multiple species, we use ZMIZ1.

## 2 *Zmiz1* in developmental biology

### 2.1 *Zmiz1* and embryogenesis: dynamic ZMIZ1 expression across germ layers and developing organ systems

Embryonic development is a highly regulated process involving crucial stages that encompass cell type specification, differentiation, tissue formation, and organ system development. Errors during the early stages of embryogenesis can lead to congenital birth defects and developmental abnormalities that persist through adulthood. ZMIZ1 is both, broadly and dynamically expressed during embryogenesis. A spatiotemporal analysis of *Zmiz1* transcripts in mouse embryos from embryonic day 7.5 (E7.5) to mid-gestation revealed expression in all three primary germ layers: the ectoderm, endoderm, and mesoderm, indicating that *Zmiz1* is active at the earliest stages of organogenesis (Rodriguez-Magadán et al., 2008), and it may contribute to the formation of multiple organ systems derived from these germ layers. At mid-gestation, *Zmiz1* expression is also detected in the dorsal aorta and the allantois, suggesting a role in vascular development and extraembryonic tissue formation. By E9.5, *Zmiz1* expression is also detected in the neural tissue, optic vesicle, otic pit, and branchial arches, which are structures that develop into the brain structures, eyes, ears, and craniofacial features, respectively. Additionally, ZMIZ1 protein expression increases during limb morphogenesis (E10.5 to E12.5) (Rodriguez-Magadán et al., 2008).

The broad and dynamic expression pattern of *Zmiz1* throughout embryonic development suggests that it contributes to the formation and maturation of multiple organ systems, including the nervous, vascular, craniofacial, and musculoskeletal systems. Consistent with these developmental expression domains, pathogenic variants or dysregulation of *ZMIZ1* have been associated with a wide spectrum of human disorders, including neurodevelopmental and neurological diseases, vascular abnormalities, cancer and benign neoplasms, gastrointestinal disorders, and congenital musculoskeletal malformations (Figure 3). These functions and disease associations are discussed in detail in Sections 1–5.

**FIGURE 3:**
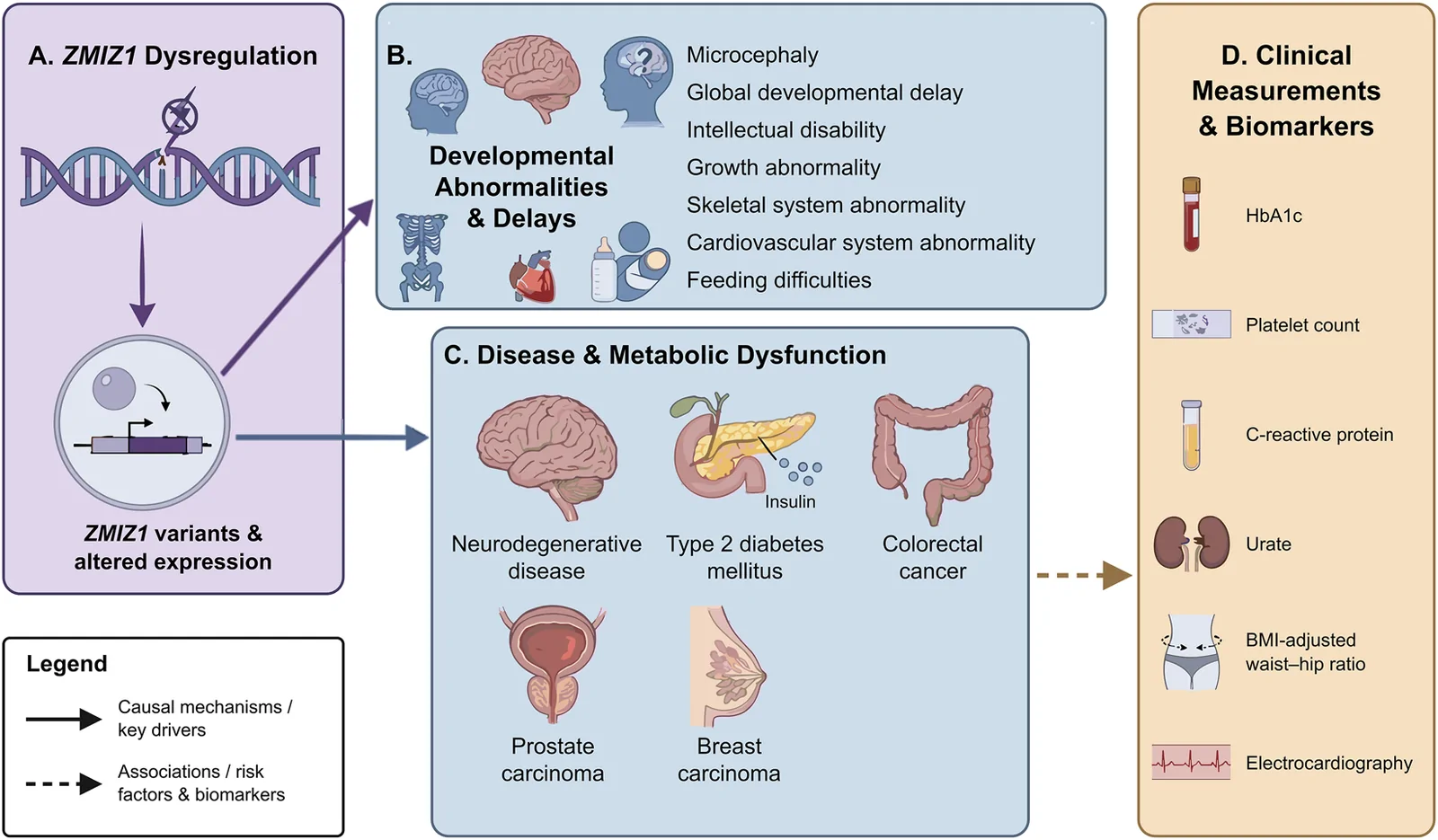
ZMIZ1 dysregulation links to a spectrum of developmental abnormalities, diseases, and clinical biomarkers. This figure reorganizes the ZMIZ1 disease-association landscape: originally retrieved as a flat therapeutic-area → disease → measurement network from the Open Targets Platform (platform.opentargets.org) into a curated conceptual model that groups outcomes by biological category and distinguishes causal drivers from statistical associations. Arrow style encodes the nature of the evidence: solid arrows denote relationships supported by causal or mechanistic evidence (“Causal Mechanisms/Key Drivers”), whereas dashed arrows denote genetic associations, risk factors, and biomarker correlations **(A)** The upstream node: pathogenic ZMIZ1 variants and altered ZMIZ1 expressions constitute the shared molecular perturbation from which downstream phenotypes emanate **(B)** Developmental abnormalities and delays caused by pathogenic variants, as defined in the ZMIZ1 syndromic neurodevelopmental disorder; microcephaly, global developmental delay, intellectual disability, growth abnormality, skeletal and cardiovascular system abnormalities, and feeding difficulties, connected by a solid arrow reflecting direct genetic causation **(C)** Diseases detected in adult stage in which ZMIZ1 is implicated across organ systems (e.g., neurodegenerative disease, type 2 diabetes, colorectal, prostate, and breast carcinoma), with evidence strength varying by condition from experimentally supported roles to GWAS-level associations **(D)** Quantitative traits and biomarkers associated with ZMIZ1 variation through genetic association and expression data (HbA1c: glycosylated hemoglobin, platelet count, C-reactive protein, urate, BMI-adjusted waist-hip ratio, and electrocardiography/PR interval), connected by dashed arrows to indicate associative rather than causal relationships. Underlying associations were curated from Open Targets evidence integrating genetic association, RNA expression, text-mining, and pathway/systems-biology data. Adapted and redesigned from platform.opentargets.org; created with BioRender.com.

### 2.2 ZMIZ1 as a transcriptional regulator of blood and lymphatic vascular development

Early investigations into the biological role of ZMIZ1 reveal its importance in early embryogenesis, specifically in the development of the vascular system (Beliakoff et al., 2008). Deletion of *Zmiz1* in mice halted development around embryonic day 10.5, accompanied by significant defects in restructuring of the yolk sac’s vascular network. In the *Zmiz1* knockout mice, the vessels in the branchial arches and dorsal aorta were poorly defined, heart morphology was abnormal, and yolk sacs were disorganized, lacking typical branching patterns of vessels (Beliakoff et al., 2008). Importantly, the expression of important embryonic vascular development genes, such as GATA-2, GATA-3, PPARγ, AP-2γ, and BMP8β, and key extraembryonic vascular development genes, such as Fra-1, was significantly reduced (Beliakoff et al., 2008), highlighting *Zmiz1*’s role in the transcriptional control of vascular development genes.

In zebrafish, the *Zmiz1* homolog, *Zmiz1*a, regulates erythropoiesis and the expression of autophagy genes (Castillo-Castellanos et al., 2021). *Zmiz1*a mutants exhibit delayed erythrocyte maturation, mitochondrial accumulation in red blood cells, and altered expression of crucial autophagy genes: *Ulk1a, Bnip4, Atg9a, Atg12*, and *Gabarapb*. In mice, targeted deletion of *Zmiz1* specifically in endothelial cells (EC) led to embryonic lethality due to vascular abnormalities (Patel et al., 2025). Studies using the retina as a model to study vascular formation during development revealed that EC-specific *Zmiz1* deletion results in decreased retinal vascular growth, density, stability, and increased vessel regression. Loss of functional *Zmiz1* in EC impacted the formation of the vascular network in both the superficial and deep retinal plexuses, particularly reducing sprouts (Patel et al., 2025). Beyond its role in developmental vascularization, *Zmiz1* also contributes to vascular regeneration under pathological conditions. Using the oxygen-induced retinopathy model, Patel and colleagues demonstrated that endothelial *Zmiz1* is crucial for hypoxia-induced revascularization. Mechanistically, loss of *Zmiz1* in retinal EC resulted in widespread transcriptional dysregulation, including downregulation of genes essential for angiogenesis and blood vessel development, including *Esm1, Angpt2, Plaur, Cxcr4, Apln, Tgfb1*, and *Tgfb3* (Patel et al., 2025). Collectively, these findings identify ZMIZ1 as an important regulator of the transcriptional programs required for formation and maturation of erythrocytes and endothelial cells in the blood vascular system during development and revascularization following injury.

In addition to its role in the blood vascular system, ZMIZ1 also plays an important role in the lymphatic vascular system. The lymphatic system runs parallel to the blood vascular system and is important for fluid homeostasis, immune surveillance, and lipid absorption. Deletion of *Zmiz1* in lymphatic endothelial cells (LEC) has an acute effect on lymphangiogenesis (K C et al., 2024a). Loss of *ZMIZ1* in human dermal lymphatic endothelial cells (HDLECs) resulted in reduced expression of genes crucial for lymphatic vessel development. Knockdown of *ZMIZ1* in HDLECs impaired lymphatic endothelial cell migration and proliferation. LEC-specific *Zmiz1* knockout mice showed a reduced number of mesenteric valves along the lymphatic vessels (K C et al., 2024a), which are critical for the overall function of the lymphatic system. Importantly, *Zmiz1* regulates the expression of PROX1, a master transcription factor in lymphatic development, by modulating chromatin accessibility at PROX1 promoter and enhancer regions (K C et al., 2024).

This evidence underscores the crucial role of *Zmiz1* in erythrocyte development and in the development of the blood and lymphatic vascular system. In both systems, ZMIZ1 orchestrates the transcriptional programs essential during embryogenesis for vascular integrity. Although key elements of the tissue-specific transcriptional programs and signaling pathways downstream of the ZMIZ1 rheostat have been identified in each system, the ZMIZ1 tissue-specific partners activating these pathways are still under investigation. In both blood and lymphatic vascular systems, the loss of ZMIZ1 function reduces activation of their respective transcriptional programs, leading to phenotypes reflecting impaired vasculogenesis. Investigating ZMIZ1’s role in pathological vascular conditions, such as cancer, retinopathy, and lymphedema, could provide valuable insights into its potential as a therapeutic target and may shed light on its potential roles in tissue repair, regeneration, and chronic disease processes.

### 2.3 ZMIZ1 in neurodevelopment: stage-specific transcriptional programs

Neurodevelopment is a precisely orchestrated process that produces the components of the nervous system and shapes brain architecture and functional neural circuitry. These processes are essential for cognition, sensory and motor functions, learning, memory, and emotional regulation. Disruptions in these neurodevelopmental processes can lead to neurodevelopmental disorders (NDDs), including autism spectrum disorder (ASD), attention-deficit/hyperactive disorder (ADHD), and intellectual disability (ID) (Exposito-Alonso and Rico, 2022). NDDs are typically polygenic and heterogeneous, involving an interplay of genetic, epigenetic, biological, and environmental risk factors.

The cerebral cortex is one of the brain regions most strongly implicated in NDDs. Structural and functional abnormalities in cortical development and organization have been reported in individuals with ASD and ID (Garcia-Forn et al., 2020; Ma et al., 2021). Key developmental processes, including cortical neurogenesis, neuronal migration, lamination, and differentiation, are critical for establishing functional cortical circuits and have been implicated in the pathogenesis of ASD (Garcia-Forn et al., 2020). Accordingly, defining the spatial, temporal, and cell-type-specific patterns of gene expression and regulation during cortical development has become an important approach to understanding the molecular basis of NDDs (Zeisel et al., 2018; Parikshak et al., 2013; Li et al., 2018). *Zmiz1* expression in the developing cortex was first characterized in mice, where it was identified as an early developmental gene with prominent expression in callosal projection neurons (CPNs) and corticothalamic projection neurons (CThPNs) (Galazo et al., 2016). These neuron types are impacted in several NDDs. CPNs, which establish interhemispheric communication through projections across the corpus callosum, are particularly relevant to ASD and contribute to higher-order functions including social behavior and cognition (Velmeshev et al., 2019; Fame et al., 2011; Hinkley et al., 2012). Consistent with this role, abnormalities in corpus callosum development and impaired interhemispheric connectivity have been associated with ASD, ID, and other neurological disorders and may contribute to deficits in social, emotional, communicative, and cognitive functions (Paul, 2011; Paul et al., 2007; Paul et al., 2014). CThPNs form corticothalamic connections, which are involved in high-level cognitive functions such as decision-making and sensory-motor control, and their developmental abnormalities are implicated in neuropsychiatric disorders (Vaasjo et al., 2022; Fazel Darbandi et al., 2018; Clascá et al., 2016; Angulo et al., 2023). Despite the prominent expression of *Zmiz1* in these neuronal populations, the roles of ZMIZ1 in CPNs, CThPNs, and cortical development more broadly have only recently begun to be elucidated. Functional studies in mice, together with emerging evidence from individuals carrying pathogenic *ZMIZ1* variants, have begun to reveal important roles for ZMIZ1 in cortical development (Carapito et al., 2019; Cormier et al., 2025; K C et al., 2024b; K C et al., 2026; Smith, 2026).

During murine brain development, *Zmiz1* expression peaks at embryonic stages, particularly in regions such as the cortex and cerebellum, and persists at lower levels at later developmental stages (K C et al., 2024b). This developmental expression pattern is consistent with a potential role for ZMIZ1 in early neurogenesis, neuronal differentiation, and circuit formation. *Zmiz1* is expressed in various cell types within the brain, including neurons, endothelial cells, astrocytes, and oligodendrocytes, with stronger expression observed in excitatory neurons compared to inhibitory neurons (K C et al., 2024b).

Deletion of *Zmiz1* specifically in the mouse cortex (*Zmiz1*-cKO model) resulted in notable structural abnormalities, including reduced cortical surface area, midline length, cortical thickness, and neuron count, leading to microcephaly (K C et al., 2026). The reduction in cortical thickness is particularly pronounced in layer 6, which is enriched in CThPNs, suggesting that these neurons may be particularly sensitive to loss of ZMIZ1 function. The reduction in cortical size and number of neurons is due to a decrease in the number and proliferative capacity of SOX2+ apical progenitors and TBR2+ intermediate progenitors during cortical neurogenesis. These progenitors produce neurons that populate all cortical layers. Additionally, the loss of *Zmiz1* resulted in abnormal development of the corpus callosum, which is smaller and has aberrant fibers forming Probst bundles, and in abnormal development of CPN in layer 2/3, which display impaired axon outgrowth toward the corpus callosum and dendritic arborization (K C et al., 2026). These findings indicate that ZMIZ1 is required at multiple stages of cortical development, influencing both the generation of cortical neurons and the subsequent maturation and connectivity of specific neuronal populations.

Consistent with these structural abnormalities, *Zmiz1*-cKO mice exhibit alterations in several behavioral domains, including locomotor activity, anxiety-like behavior, communication, and social interaction, with notable sex-dependent differences (K C et al., 2026). Male *Zmiz1*-cKO mice exhibited increased locomotor activity, increased anxiety-like behavior, and communication deficits characterized by reduced ultrasonic vocalizations and a more limited repertoire of call types. Both male and female mutants displayed abnormal social interactions; however, the nature of these deficits differed between sexes. Male mice showed an increased preference for social novelty compared with control animals, whereas female mice lacked a preference for social novelty (K C et al., 2026). Importantly, these behavioral phenotypes recapitulate selected, sex-dependent aspects of the neurobehavioral phenotypes reported in individuals with ZMIZ1-related neurodevelopmental disorders, as discussed in the following section. These results also suggest that dysfunction in other neural cell types and brain regions, beyond cortical excitatory neurons, likely contributes to the full spectrum of neurological manifestations associated with ZMIZ1 syndrome.

Transcriptome analyses of cortices from embryonic, neonatal, and juvenile stages revealed that ZMIZ1 regulates distinct gene-expression programs across neurodevelopment (K C et al., 2026). At embryonic and neonatal stages, the loss of *Zmiz1* preferentially affected pathways associated with neurogenesis, neuronal differentiation, axon development, and axon guidance, with reduced expression of genes including *Lhx2, Auts2, Epha5, Efnb2,* and *Tbr1*. In contrast, at juvenile stages, the transcriptional consequences of *Zmiz1* loss were dominated by alterations in genes involved in synaptic transmission, suggesting that the requirement for ZMIZ1 changes as cortical circuits mature. Confirmation via chromatin immunoprecipitation and functional luciferase assays confirmed the identification of a substantial number of differentially expressed genes as transcriptional targets downstream of ZMIZ1 (K C et al., 2026). Importantly, differentially expressed genes in *Zmiz1*-cKO cortices showed significant overlap with genes implicated in ASD risk in the SFARI database, providing a potential molecular link between ZMIZ1-dependent transcriptional regulation and pathways associated with neurodevelopmental disorders (K C et al., 2026). Functional rescue experiments provided additional evidence that altered transcriptional regulation contributes directly to the cortical phenotypes caused by ZMIZ1 loss. *In vivo* overexpression of *Efnb2* in CPNs partially rescued the impaired dendritic growth observed in *Zmiz1*-cKO mice (K C et al., 2026).

These findings support a model in which ZMIZ1 regulates distinct transcriptional programs at different stages of cortical development, with early functions associated predominantly with neurogenesis, neuronal differentiation, and axonal development and later functions associated with synaptic maturation and transmission. This stage-dependent regulatory activity is consistent with the proposed role of ZMIZ1 as a transcriptional rheostat, whereby its transcriptional output is determined by cellular context, protein abundance, and interactions with different transcriptional partners.

An important question emerging from this model is how ZMIZ1 switches between these developmental programs. One possibility is that partner-dependent interactions contribute to this transition. For example, interactions between ZMIZ1 and NOTCH1, a key regulator of neural progenitor proliferation and cell fate, could contribute to the transcriptional programs governing early cortical neurogenesis. At later developmental stages, interactions with androgen and estrogen receptors, could potentially influence transcriptional programs involved in synaptic maturation and circuit function and thereby contribute to sex-dependent phenotypic differences. These mechanisms remain to be directly established in the developing cortex and should therefore be considered as testable models rather than established pathways. More broadly, the developmental expression and activity of ZMIZ1 may provide an additional layer of regulation (dose-dependent axis), with changes in ZMIZ1 abundance and availability influencing its interactions with distinct transcriptional partners and consequently shaping its stage-specific transcriptional output.

Collectively, all evidence available indicates that in the context of neurodevelopment, the loss of ZMIZ1 function reduced the activation of transcriptional programs required for normal cortical development, resulting in abnormalities in neural progenitor proliferation, neuronal differentiation, axonal growth, cortical connectivity, and synaptic function.

## 3 *Zmiz1* in neurological disorders

### 3.1 Pathogenic ZMIZ1 variants and syndromic neurodevelopmental disorders

The potential involvement of ZMIZ1 in NDDs was first suggested by the identification of a *de novo* translocation involving ZMIZ1 by Córdova-Fletes and colleagues in 2015 (Córdova-Fletes et al., 2015). The translocation resulted in the formation of a ZMIZ1/PRR12 reciprocal fusion transcript in a patient with ID and neuropsychiatric alterations, including anxiety, impaired visual contact and social communication, reduced attention span, and memory impairment. Subsequently, Phetthong and colleagues reported another patient in whom a *ZMIZ1* variant occurred in combination with a chromosomal microdeletion involving *OTUD6B* and was associated with ID and developmental delay (Phetthong et al., 2021) Schluth-Bolard and colleagues analyzed the functional and phenotypic consequences of balanced chromosomal rearrangements (BCRs) in patients with developmental disorders and identified various breakpoints within *ZMIZ1*, which resulted in different fusion genes (Schluth-Bolard et al., 2019). Although such rearrangements are rare, these observations provided early evidence that disruption of the *ZMIZ1* locus can contribute to developmental phenotypes, including developmental delay, ID, and speech impairment.

Additional evidence linking *ZMIZ1* to NDDs emerged from genomic studies of ASD. A whole-genome sequencing (WGS) study identified *ZMIZ1* as a candidate ASD risk gene using a novel statistical framework designed to identify disease-associated variants and reported a single-nucleotide variant within a *ZMIZ1* enhancer as a potential genetic risk factor for ASD (Liu et al., 2018). More direct evidence came from Carapito and colleagues, who identified a cohort of 19 patients with pathogenic *ZMIZ1* variants identified as causal of a syndromic neurodevelopmental disorder characterized by ID, ASD, developmental delay, impaired language acquisition, behavioral abnormalities, growth abnormalities, feeding difficulties, microcephaly, craniofacial dysmorphism, and mild skeletal anomalies affecting the hands and feet (Carapito et al., 2019). This phenotype was subsequently designated neurodevelopmental disorder with dysmorphic facies and distal skeletal anomalies (NEDDFSA) (Li et al., 2025) or ZMIZ1-associated neurodevelopmental disorder (Kruidenier et al., 2025). Subsequent reports describing additional individuals and *ZMIZ1* variants, most of which were *de novo*, have expanded the phenotypic spectrum and further strengthened the association between pathogenic *ZMIZ1* variants and syndromic NDDs (Latchman et al., 2020; Li et al., 2025; Mahjani et al., 2021; Zhou X. et al., 2022; Brea-Fernández et al., 2022; He et al., 2024; Lu et al., 2022; Bartolomaeus et al., 2023; Alghamdi et al., 2022; Kruidenier et al., 2025; Rogan et al., 2026; De Rosa and Kalantari, 2026; Nat et al., 2026).

The association between *ZMIZ1* and ASD is also supported by genomic studies of affected individuals and families. Whole-exome sequencing of *postmortem* brain tissue from individuals with ASD identified *ZMIZ1* among genes harboring potentially pathogenic variants, providing additional evidence for its involvement in ASD-related molecular pathways (Nat et al., 2026; Woodbury-Smith et al., 2022).

Interestingly, large-scale exome-sequencing studies of families affected by ASD and developmental disorders have demonstrated an increased burden of *de novo* protein-truncating and other loss-of-function variants in genes that are highly expressed during human brain development, including genes involved in transcription regulation and chromatin remodeling, such as *ZMIZ1* (Iossifov et al., 2014; McRae, 2017). Given the established contribution of chromatin-regulatory genes to ASD risk and the relatively recent recognition of pathogenic *ZMIZ1* variants as a cause of syndromic NDDs, the contribution of *ZMIZ1* variation to ASD and related phenotypes may have been underrecognized.

Collectively, these findings establish *ZMIZ1* as an important regulator of neurodevelopment and provide converging genetic evidence linking pathogenic *ZMIZ1* variants to NDDs, including ID, ASD, developmental delay, and associated craniofacial and skeletal abnormalities. The phenotypic complexity observed in individuals with *ZMIZ1*-associated NDDs is consistent with the diverse developmental functions of ZMIZ1 revealed by experimental studies in mice. In particular, the requirement for ZMIZ1 to regulate multiple transcriptional programs at multiple stages of cortical development provides a potential mechanistic framework for understanding the broad range of neurological phenotypes observed in affected individuals. However, the consequences of pathogenic *ZMIZ1* variants are unlikely to be restricted to the cerebral cortex. The molecular and cellular mechanisms underlying the craniofacial, skeletal, growth, feeding, and other systemic manifestations of NEDDFSA remain poorly understood and represent important areas for future investigation.

Beyond the established association between *ZMIZ1 de novo* variants and syndromic NDDs, *ZMIZ1* has also been implicated in other disorders affecting the nervous system, although the strength of evidence varies substantially among these conditions. One such association is Hirschsprung disease (HD), a congenital disorder characterized by impaired development of the enteric nervous system and consequent intestinal obstruction. A reported individual carrying a *de novo* truncating *ZMIZ1* variant presented with HD in addition to developmental and cognitive delay and several dysmorphic features characteristic of NEDDFSA (Valind et al., 2021). No additional chromosomal abnormalities or established genetic risk variants for HD were identified in this individual. Given that ZMIZ1 is expressed in progenitors of the enteric nervous system and in a subpopulation of enteric cholinergic neurons (Zeisel et al., 2018). These observations led the authors to propose that the enteric phenotypes may be associated with *ZMIZ1* haploinsufficiency and that HD could represent an additional manifestation of *ZMIZ1*-associated NDDs. However, further individuals and functional studies will be required to determine whether this association is reproducible and whether the penetrance of enteric phenotypes depends on the specific molecular consequence of individual *ZMIZ1* variants (Valind et al., 2021).

*ZMIZ1* has also been implicated in depression, although evidence for a direct relationship remains limited. Wei and colleagues identified *Zmiz1* as a potential regulatory gene in a mouse model of depression based on differential gene-expression analyses of the dentate gyrus and anterior cingulate cortex (Wei et al., 2021). Their analysis suggested that *Zmiz1* may regulate multiple hub genes that were differentially expressed in the dentate gyrus, implicating ZMIZ1-dependent transcriptional regulation in molecular pathways associated with depression. These findings provide a hypothesis-generating link between ZMIZ1 and depressive phenotypes but do not establish that altered ZMIZ1 function is a primary cause of depression. Additional genetic, cellular, and functional studies will be necessary to determine whether ZMIZ1 contributes directly to the pathophysiology of depression.

Overall, pathogenic *ZMIZ1* variants are increasingly recognized as causes of syndromic neurodevelopmental disorders characterized by a variable combination of ASD, ID, developmental delay, behavioral abnormalities, and craniofacial and skeletal manifestations. The proposed framework in which ZMIZ1 functions as a partner-directed transcriptional rheostat is consistent with the transcriptional dynamics and cellular functions of ZMIZ1 identified in the developing mouse cortex, and with the complexity of neurological phenotypes observed in individuals with *ZMIZ1*-associated NDDs.

### 3.2 ZMIZ1 *in Neurodegeneration and aging-related neuronal dysfunction*

In addition to ZMIZ1’s association with neurodevelopmental disorders, *ZMIZ1* has recently been associated with neurodegenerative disease and aging-related neuronal dysfunction. Genetic and transcriptomic studies have linked *ZMIZ1* to Lewy body disease (LBD) (Hou et al., 2025) and to molecular pathways associated with neuronal aging and senescence (Chen et al., 2025; Deng et al., 2026). In a GWAS study of neuropathological traits, significant associations with phosphorylated serine-65 ubiquitin (p-S65-Ub), a marker of mitochondrial damage and mitophagy, were identified for both *APOE* rs429358 and *ZMIZ1* rs6480922 (Hou et al., 2025). Notably, the effects of these variants were directionally distinct: the *APOE4*-associated allele was associated with increased p-S65-Ub levels, whereas the *ZMIZ1* rs6480922 variant was associated with lower p-S65-Ub levels (Hou et al., 2025).

P-S65-Ub accumulates on damaged mitochondria following PINK1 activation and phosphorylation of ubiquitin, and is widely used as a molecular marker of impaired mitochondrial health and altered mitophagy (Chen et al., 2023; Fiesel and Springer, 2015). Because mitochondrial dysfunction and defective proteostasis are central features of several neurodegenerative diseases, including Parkinson’s disease and LBD, variation in genes influencing these pathways may contribute to differences in disease susceptibility or neuropathological burden. In the context of LBD, the *ZMIZ1* rs6480922 variant was associated with greater brain weight and lower levels of several neuropathological measures, including α-synuclein burden, senile plaque density, and neurofibrillary tangle density (Hou et al., 2025). Importantly, the effects of the *ZMIZ1* variant on p-S65-Ub and neuropathological measures were modulated by *APOE4* carrier status, raising the possibility of an interaction between ZMIZ1-and APOE-dependent pathways in neurodegenerative disease (Hou et al., 2025). However, the mechanisms underlying these associations remain unknown, and the available evidence does not establish whether the *ZMIZ1* variant directly alters mitochondrial health or whether the observed relationships reflect indirect or context-dependent effects.

Additional evidence for a role of ZMIZ1 in neuronal homeostasis comes from studies of Alzheimer’s disease models. In basal forebrain cholinergic neurons from 5xFAD mice, *Zmiz1* was identified as a regulator of gene networks involved in cholesterol and lipid metabolism, DNA damage repair, and death receptor signaling (Chen et al., 2025). These pathways are of particular interest because alterations in lipid metabolism, mitochondrial function, DNA damage responses, and cellular stress are increasingly recognized as interconnected mechanisms contributing to neuronal dysfunction and neurodegeneration. The involvement of *Zmiz1* in these pathways is also consistent with the broader role of ZMIZ1 as a context-dependent transcriptional regulator. However, whether ZMIZ1 directly regulates these pathways in human neurodegenerative disease, and whether altered ZMIZ1 activity contributes to disease initiation or progression, remains to be determined.

The potential relationship between ZMIZ1 and neurodegeneration is particularly intriguing in light of evidence linking *Zmiz1* to aging-related neuronal senescence (Chen et al., 2025; Deng et al., 2026). Together, these findings raise the possibility that ZMIZ1 may influence neuronal resilience through transcriptional programs controlling lipid metabolism, mitochondrial homeostasis, DNA damage responses, and cellular survival. Such functions could provide a mechanistic connection between ZMIZ1-dependent transcriptional regulation and the accumulation of neuropathological changes during aging. Nevertheless, the current evidence is predominantly associative, and additional genetic and functional studies are needed to determine whether ZMIZ1 represents a causal modifier of neurodegenerative disease or instead serves as a marker of disease- or age-dependent cellular states.

Future studies should determine how ZMIZ1 variation influences mitochondrial health, proteostasis, lipid metabolism, and neuronal survival, and whether these effects depend on cellular context or interaction with other disease-associated factors such as APOE4. Defining the molecular consequences of the rs6480922 variant and determining whether it alters ZMIZ1 expression, protein activity, or interactions with transcriptional partners will be particularly important. Such studies may reveal whether ZMIZ1-dependent pathways represent potential therapeutic targets in LBD and related neurodegenerative disorders.

## 4 Zmiz1 in immune system and related diseases

### 4.1 ZMIZ1 in T-cell development and leukemogenesis

T cells play a central role in adaptive immunity, and their development requires coordinated regulation of lineage specification, differentiation, proliferation, and survival. Disruption of these processes can contribute to immunodeficiency, autoimmune disease, and hematologic malignancies.

During normal T-cell development, NOTCH1 signaling is a major regulator of T-lineage specification, maturation, and survival (Hozumi et al., 2008; Han et al., 2002). ZMIZ1 exhibits a similar developmental expression pattern to that of NOTCH1, with highest expression in early T-lineage progenitors (ETPs) and progressively lower levels as T cells mature (González-García and Toribio, 2009). Both ZMIZ1 and NOTCH1 are required for proper ETP specification (Wang et al., 2018). Mechanistically, ZMIZ1 directly interacts with NOTCH1 through its TPR domain and co-occupies regulatory elements of a subset of NOTCH1 target genes, including the oncogene *Myc* (Wang et al., 2018; Amarante-Mendes et al., 2022). Interestingly, this interaction is developmentally regulated: ZMIZ1 attenuates NOTCH1 activity during the earliest stages of T-cell development but enhances NOTCH1 signaling after lineage commitment to support T-cell maturation (Wang et al., 2018). ZMIZ1 is also recruited during thymic stress, where it promotes T-cell regeneration (Wang et al., 2018), highlighting its context-dependent role in T-cell biology.

This relationship between ZMIZ1 and NOTCH1 is particularly relevant to T-cell acute lymphoblastic leukemia (T-ALL), an aggressive malignancy in which activating *NOTCH1* mutations occur in approximately 60% of cases (Rakowski et al., 2013). Notch signaling is essential for maintaining intestinal homeostasis. Therapeutic inhibition of NOTCH signaling via pan-Notch inhibitors has been challenging because broad pathway inhibition disrupts intestinal homeostasis and causes significant gastrointestinal toxicity (Pinnell et al., 2015). Furthermore, broad inhibition of Notch signaling may have unintended oncogenic consequences, as Notch can also function as a tumor suppressor in certain cellular contexts (Pinnell et al., 2015). Importantly, activating *NOTCH1* mutations alone are insufficient to induce T-ALL leukemia in mouse models, indicating that additional factors cooperate with NOTCH1 to drive leukemogenesis.

ZMIZ1 has emerged as an important mediator of NOTCH1-driven T-ALL. *Zmiz1* depletion via knockdown in malignant lymphoblastic cells delayed tumor growth, reduced cell-cycle progression, and decreased glucose consumption (Rakowski et al., 2013). Interestingly, ZMIZ1 deletion inhibited leukemogenesis, without causing the intestinal toxicity, weight loss, or myeloproliferative disease observed with Notch1 inhibition (Rakowski et al., 2013). Combining ZMIZ1 depletion with NOTCH inhibitors or corticosteroids further impaired leukemic growth and increased treatment sensitivity (Rakowski et al., 2013). These findings suggest that ZMIZ1 may represent a therapeutically relevant vulnerability downstream of NOTCH1, that could avoid the negative consequences of Notch inhibitors while combating leukemic growth.

As discussed above, *MYC* is a well-established oncogene frequently dysregulated in many cancers, including T-ALL leukemia, and is regulated by ZMIZ1. ZMIZ1 and NOTCH1 cooperate to activate the MYC transcriptional program (Rakowski et al., 2013). Mechanistically, ZMIZ1 directly interacts with NOTCH1 through its TPR domain, facilitating the recruitment of NOTCH1 to the 3′MYC enhancer. Mutations in ZMIZ1’s TPR domain disrupt the ZMIZ1–NOTCH1 interaction, impair MYC activation, block the double-negative (DN)-to-double-positive (DP) transition during T-cell maturation, and suppress leukemic cell proliferation (Wang et al., 2018). These findings identify ZMIZ1’s TPR domain as a critical mediator of ZMIZ1–NOTCH1-dependent MYC activation and suggest that disrupting this interaction could attenuate NOTCH1/MYC-driven leukemogenesis. Supporting the feasibility of pharmacological targeting, small-molecule ZMIZ1 inhibitors have recently demonstrated on-target activity and antileukemic effects in murine acute myeloid leukemia models and AML organoids (Li et al., 2026). Further studies are needed to establish their specificity, mechanism of action, and therapeutic window.

In addition to its transcriptional role in T-ALL, *ZMIZ1* has been identified as a fusion partner of the oncogene *ABL1* in several leukemia cases (Soler et al., 2008; Liu et al., 2023; Boer et al., 2017). ABL1 encodes a tyrosine-protein kinase (TK), and chromosomal rearrangements that generate constitutively active ABL1 fusion proteins can promote aberrant signaling and malignant proliferation (Amarante-Mendes et al., 2022). Identification of candidate fusion partner genes for ABL1 is valuable in both the diagnosis and treatment of leukemias. A *ZMIZ1-ABL1* fusion was identified in a Dutch/German pediatric B-cell precursor acute lymphoblastic leukemia (BCP-ALL) cohort, with the predicted kinase activity suggesting potential sensitivity to tyrosine kinase inhibitors (TKIs) (Boer et al., 2017). A similar fusion was reported in an 18-month-old girl with B-cell ALL; the predicted fusion protein contained the ABL1 kinase domain and the proline-rich region of ZMIZ1, potentially enabling constitutive kinase activity, and it was suggested that this patient may be eligible for treatment with the TKI Imatinib (Soler et al., 2008). In another case, a 60-year-old man with myelomonocytic leukemia carrying a *ZMIZ1-ABL1* fusion achieved hematologic remission following treatment with the TKI Dasatinib, with no detectable fusion after 1 year (Liu et al., 2023). Although these reports are limited to individual cases, they highlight the potential clinical relevance of identifying *ZMIZ1-ABL1* fusions and suggest that affected patients may benefit from TKI-based treatment.

Collectively, ZMIZ1 contributes to leukemia through at least two distinct mechanisms. In T-ALL, ZMIZ1 functions as a context-dependent transcriptional cofactor that modulates NOTCH1 and supports MYC-driven leukemic programs. In other leukemias, *ZMIZ1-ABL1* rearrangements generate potentially oncogenic fusion kinases. These findings further illustrate the functional versatility of ZMIZ1 and the importance of its cellular context and protein interactions in determining disease-associated functions. Targeting the ZMIZ1–NOTCH1 interface or identifying actionable *ZMIZ1-ABL1* fusions may therefore represent promising therapeutic strategies in selected leukemia subtypes.

### 4.2 Reduced ZMIZ1 expression as a shared risk factor across autoimmune diseases

Autoimmune disorders are characterized by abnormal immune responses against self-antigens, leading to tissue damage and dysfunction. Various studies report strong heritability, indicating a need to explore common genetic pathways among these disorders (Cooper and Stroehla, 2003). Several studies have highlighted the role of *Zmiz1* in autoimmune disorders, including multiple sclerosis (MS), vitiligo, and inflammatory bowel disease (IBD).

#### 4.2.1 Reduced ZMIZ1 expression and vitamin D–Responsive risk in multiple sclerosis

Multiple sclerosis (MS) is a chronic autoimmune disease that primarily affects the central nervous system. In MS, the immune system triggers an inflammatory response that targets the myelin sheaths surrounding axons, resulting in oligodendrocyte damage, demyelination, and ultimately neurodegeneration. GWAS have identified numerous genetic risk factors, while environmental influences, including Epstein–Barr virus (EBV) infection and Vitamin D exposure, are also strongly implicated in disease susceptibility. Despite these advances, the events initiating the disease and controlling its progression remain elusive, limiting improvements in disease diagnosis, prognosis, and the development of effective therapies.

*ZMIZ1* first association of ZMIZ1 with MS comes from GWAS in the blood of MS patients and controls, which identified *ZMIZ1* as MS risk gene (Parnell et al., 2014). Another study identified two SNPs, rs1250542 and rs1250550, in the *ZMIZ1* locus strongly associated with MS (Fewings et al., 2017) and identified a significant reduction of ZMIZ1 expression in immune cells of MS patients, particularly in plasmocytic dendritic cells. In three different patient cohorts, low *ZMIZ1* expression conferred an MS risk with an odds ratio of 7.9, which is comparable to major MS risk factors, such as EBV (Fewings et al., 2017). *ZMIZ1* expression positively correlated with genes *ZFP36L2, JARID2*, and *STAT3* and negatively correlated with *RPS6KB1, CD48,* and *BATF*, many of which play a crucial role in the immune response (Fewings et al., 2017). Also, ZMIZ1 expression was found to be negatively correlated with antibodies to EBV, which are associated with MS pathogenesis. Notably, ZMIZ1 expression significantly increased in MS patients under two MS-treatments (fingolimod and dimethyl fumarate) compared to untreated MS patients. In addition, this study found evidence that ZMIZ1 is regulated by Vitamin D (Fewings et al., 2017; Zarzuelo Romero et al., 2021). This observation is important, since MS is considered a latitude-dependent autoimmune disorder (LDADs), and provides a potential mechanistic link between MS and ZMIZ1 expression.

Vitamin D deficiency increases MS risk, and genes that control Vitamin D metabolism are also risk factors for LDADs, including MS (Ong et al., 2020). Vitamin D Receptor (VDR) binding sites are enriched in near LDADs genes, MS risk genes, and were identified near *ZMIZ1* in immune cells (Ong et al., 2020; Booth et al., 2016). Vitamin D upregulates ZMIZ1 expression in tolerogenic dendritic cells, but not in inflammatory dendritic cells (Fewings et al., 2017). Methylome analysis revealed differences in methylation in *ZMIZ1* MS risk SNP rs1250551 near the VDR binding site in *ZMIZ1*. Methylation at this location was lower in MS patients compared to healthy controls. However, methylation was not correlated with gene expression in the blood (Ong et al., 2020). The interpretations of the association between locus methylation and gene expression must be very cautious. Any association with expression may be context-dependent and may need very large cohorts for statistical power to detect it. The implication of this risk SNP and disease association is that methylation of this VDR binding site in a Vitamin D-regulated gene affects disease pathogenesis, presumably through effects on transcription factor binding.

This *ZMIZ1* phenotype in MS, characterized by altered gene expression, immune cell profiles, methylation patterns, and Vitamin D responsiveness, suggests its prognostic potential. Investigating molecular mechanisms by which *ZMIZ1* modulates immune responses, focusing on its interaction with Vitamin D signaling and its role in dendritic cell function and its expression as a biomarker for MS progression and therapeutic response, may enhance treatment approaches and improve prognostic accuracy.

#### 4.2.2 ZMIZ1 regulatory variants and melanocyte survival in vitiligo

Vitiligo is a skin disorder characterized by the dysfunction of melanocytes in the epidermis. The prevalence of vitiligo in the United States is estimated to be between 0.76% and 1.11% (Gandhi et al., 2022). While not considered to be life-threatening, it carries a high psychological burden and costs the US public healthcare system an estimated $175 million (Hadi et al., 2020). Vitiligo is a “complex disease” reflecting a combination of genetic and environmental factors with links to other autoimmune diseases such as Crohn’s disease, inflammatory bowel disease, multiple sclerosis, and type 1 diabetes (Hu and Wang, 2023). Over half of the vitiligo loci encode immunoregulatory proteins, reflecting its autoimmune nature (Spritz and Santorico, 2021). While there are multiple theories as to the cause of vitiligo, such as loss and degradation of melanocytes due to autoimmunity, cytotoxic metabolites, and oxidative stress, the molecular pathways and processes that lead to melanocyte degeneration remain unclear (Chen et al., 2021) (Liu et al., 2023).

GWAS study of 5,000 individuals in the Chinese Han population found *ZMIZ1* to have a significant association with vitiligo (Quan et al., 2010). Fine-mapping analysis identified rs1408944 as a novel single nucleotide polymorphism in the *ZMIZ1* locus as a risk factor for vitiligo independent of other known autoimmune disease-associated SNPs in *ZMIZ1* (Sun et al., 2014). This SNP is in an open chromatin intronic region of *ZMIZ1,* where binding sites for at least six transcription factors have been identified by ChIP-seq analysis. Therefore, it is likely that rs1408944 confers risk for vitiligo by altering transcription factor binding and *ZMIZ1* expression. The authors hypothesized that rs1408944 influences the development of vitiligo by interrupting melanocyte development and survival through affecting the transcriptional regulation of ZMIZ1 and perhaps affects the ZMIZ1-PIAS-MITF pathway. *PIAS3* and *ZMIZ1* are both members of the PIAS family. PIAS3 has been found to inhibit the transcriptional activity of *MITF*, a key regulator of melanocyte development, function, and survival (Sun et al., 2014). However, more information regarding how ZMIZ1 expression is affected by this SNP, and the interaction between elements of ZMIZ1-PIAS-MITF pathway is necessary to interpret the functional impact of this SNP in vitiligo.

More direct evidence implicating *ZMIZ1* in vitiligo comes from studies with human skin biopsies and *in vitro* human melanocyte cell lines, *PIG1* and *PIG3V* (Lu et al., 2024). ZMIZ1 expression was reduced in vitiligo skin samples. *In vitro* experiments showed that decreased ZMIZ1 expression inhibits melanocyte proliferation and migration, reduces their viability, and increases apoptotic rates. ZMIZ1-knockdown also decreased the activation of the melanogenesis-associated mTOR/AKT/GSK-3β pathway, which results in lower expression of melanin synthesis proteins, tyrosine-related protein-1 and tyrosinase, and an increase in reactive oxygen species (ROS) content. These results indicate ZMIZ1’s role in the pathogenesis of vitiligo may be mediated via mTOR/AKT/GSK-3β signaling pathway, and that decreased ZMIZ1 in melanocytes is likely an important risk factor. Together, these studies suggest that ZMIZ expression regulation in melanocytes is affected in vitiligo, and suggest that decreased levels confer risk through a yet unclear mechanistic pathway.

#### 4.2.3 ZMIZ1 as a T cell–restricted eQTL and Crohn’s disease susceptibility locus

Inflammatory bowel diseases (IBDs) are chronic heritable autoimmune diseases, characterized by inflammation of the gastrointestinal tract in the form of Crohn’s disease and ulcerative colitis. Recent research has revealed that there are over 100 loci significantly associated with IBDs (Cho and Brant, 2011). Among these, the *ZMIZ1* locus has emerged as a recurrently implicated susceptibility region. GWAS and expression quantitative trait locus (eQTL) analyses have identified *ZMIZ1* as a susceptibility gene for CD, suggesting that genetic variation within this locus influences immune-related gene expression programs associated with disease risk (Dubois et al., 2010; Alegbe et al., 2026). Consistent with these findings, GWAS focused on early-onset IBD, which has a stronger genetic contribution, further supported an association between *ZMIZ1* and higher disease risk (Western Regional Research Alliance for Pedi atric IBD et al., 2009). Jakobsen and colleagues also identified *ZMIZ1* among loci significantly associated with IBD susceptibility in a large cohort of Danish children (Jakobsen et al., 2014). Interestingly, this pediatric IBD study identified the SNP rs1250550 within *ZMIZ1* as being associated with reduced susceptibility to Crohn’s disease (Jakobsen et al., 2014). Furthermore, colocalization analyses demonstrated overlap between a Crohn’s disease-associated locus and a *ZMIZ1* eQTL specifically in undifferentiated T cells (Soskic et al., 2022), suggesting that *ZMIZ1* may contribute to Crohn’s disease pathogenesis through modulation of T-cell function.

Collectively, these studies provide associative evidence of *ZMIZ1* contribution to MS, vitiligo and IBD, the strongest functional association being with MS. The effects of ZMIZ1 are cell-context dependent, and the impact of genetic variants in *ZMIZ1* transcription regulation is still unclear. However, in the context of MS and vitiligo, the available evidence suggests that downregulation of ZMIZ1 is associated with higher risk. The potential contribution of *ZMIZ1* variants to IBD risk is still unclear.

## 5 Zmiz1 in metabolic disorders

### 5.1 ZMIZ1 dosage in β-cell function and diabetes risk

Diabetes is a complex metabolic disorder characterized by hyperglycemia, insulin resistance, and impaired insulin secretion. Both genetic and environmental factors contribute to its etiology and progression. Diabetes is considered to be one of the biggest epidemics of the 21st century and is a gateway disease, leading to blindness, heart disease, kidney failure, and stroke.

Multiple GWAS have identified several susceptibility gene loci for Type-2 Diabetes (T2D), including *ZMIZ1*. Andersen et al. (2014) investigated the role of genetic variants in predisposing individuals to adult-onset autoimmune diabetes and found that *ZMIZ1* variant rs12571751 (p = 4.1 × 10^−5^) is associated with T2D susceptibility (Andersen et al., 2014). A genome-wide meta-analysis combining data from approximately 150,000 individuals has found a strong association between the *ZMIZ1* locus and T2D risk (P = 1 × 10^−10^) (the DIAbetes Genetics Replication And Meta-analysis DIAGRAM Consortium et al., 2012). Expression quantitative trait locus (eQTL) analyses in human pancreatic islets further implicated the involvement of *ZMIZ1* in glucose homeostasis and islet function (Van et al., 2015). Additionally, genome-wide SNP genotyping identified candidate markers, such as *ZMIZ1,* mediating susceptibility to end-stage renal disease attributed to Type 1 Diabetes (Craig et al., 2009).

Direct functional evidence of ZMIZ1’s role in islet biology and insulin secretion arises from ZMIZ1 gain and loss of function. When *ZMIZ1* is overexpressed, insulin secretion is reduced due to a decrease in the release of insulin-containing vesicles. *ZMIZ1* expression directly affects the exocytosis mechanism of insulin secretion (Van et al., 2015). *ZMIZ1* also plays a crucial role in the function of mature islet beta-cells (β-cells) and their expansion during high-fat feeding (Alghamdi et al., 2022). To determine the impact of *ZMIZ1* on glucose homeostasis, β-cells specific *Zmiz1* knockout mice (*Zmiz1-βKO*) were generated and characterized. These mice exhibited both glucose intolerance and impaired insulin secretion (Alghamdi et al., 2022). Additionally, in *Zmiz1-βKO* mice, loss of *Zmiz1* causes dysregulation in 556 genes, including those crucial to β-cells maturation, proliferation, differentiation, insulin secretion, and glucose metabolism. The seemingly paradoxical results that both gain and loss of ZMIZ1 expression reduce insulin secretion are likely explained by actions of ZMIZ1 in different mechanisms in a cell-stage-dependent manner. In mature β-cells, ZMIZ1 controls vesicle release, and in this context, high-dose ZMIZ1 is detrimental because it reduces insulin secretion. During proliferation and differentiation of β-cells, ZMIZ exerts transcriptional control of genes important for β-cell production, growth, and maturation. In this context, low-dose ZMIZ1 is also detrimental because it impairs the production and function of β-cells, which ultimately reduces insulin secretion.

Overall, *ZMIZ1* regulates several mechanisms that control differentiation and function of β-cells and insulin secretion. Precise control of ZMIZ1 dosage is therefore critical for proper β-cells function; its dysregulation is linked to the etiology of metabolic disorders such as diabetes.

## 6 *Zmiz1* and cancers

*ZMIZ1* has been implicated in the development and progression of various types of cancer, including skin carcinoma, prostate cancer, breast cancer, colorectal cancer, osteosarcoma, and glioblastoma. Given the breadth of cancers associated with ZMIZ1, and therefore the heterogeneity of cellular contexts, it is unlikely to find a unifying mechanism or pathway on which ZMIZ1 converges to contribute as an oncogene. For example, in hormone-responsive cancers, ZMIZ1 often increases transcription driven by androgen or estrogen receptors. In other tumors, ZMIZ1 activity is altered due to changes in expression, protein truncation, presence of circular or antisense RNAs, transcript usage, or epigenetic changes. These different mechanisms may lead to common cellular consequences, such as enhanced proliferation, survival, migration, invasion, autophagy, and resistance to therapy. In the following sections, we discuss evidence of ZMIZ1 involvement in different cancers, and at the end, we discuss potential context-dependent discrepancies revealed in the literature.

### 6.1 ZMIZ1 truncation produces a potential oncogenic protein in skin cancer

Nonmelanoma skin cancer (NMSC) is considered to be the most common skin cancer. Cutaneous squamous cell carcinoma (cuSCC) is a subtype of NMSC that originates from an uncontrolled proliferation of atypical epidermal keratinocytes of the skin (Fania et al., 2021). Because high-risk cuSCC carries a poor prognosis due to rapid progression, lack of biomarkers, and limited targeted therapies, exploring its genetic landscape is crucial to identify key drivers and actionable targets.

A Sleeping Beauty (SB) transposon mutagenesis screen on a *Trp53*-deficient genetic background identified *Zmiz1* as a candidate oncogene and the most significant recurrent driver of cuSCC within a broader landscape of dysregulated pathways, including NOTCH, WNT, and MAPK signaling (Aiderus et al., 2021). Analysis of transposon insertion sites found that nearly 90% of skin tumors had transposon insertions in the *Zmiz1* locus, which resulted in the overexpression of an N-terminally truncated protein. *Zmiz1* ^Δ1-185^ is more stable compared to the untruncated ZMIZ1 protein and might confer selective advantage to tumor cells (Dupuy et al., 2009; Rogers et al., 2013). Importantly, the SB-induced truncated transcript of ZMIZ1 was found to be similar to the alternative endogenous *ZMIZ1* isoform (uc001kag-2) overexpressed in human SCCs (Rogers et al., 2013). In *Zmiz1* ^Δ1-185^ double-transgenic mice, 85% developed an average of 2.7 skin tumors per mouse, demonstrating rapid tumor development without an external promoting agent (Rogers et al., 2013).

Given *Zmiz1*’s role as a transcriptional co-activator and SUMOylating promoter, the increased stability of *Zmiz1* ^Δ1-185^ may lead to excessive activity, contributing to its oncogenic potential (Rogers et al., 2013). Moreover, gene expression profiling on *Zmiz1* ^Δ1-185^ transgenic tumors showed upregulation of skin tumor susceptibility genes such as *Serpinb3a*, indicating a downstream tumorigenic pathway induced by *Zmiz1* (Rogers et al., 2013). Interestingly, all SB-induced cuSCC tumors with *Zmiz1*-mutating insertions had at least one inactivating mutation in chromatin-remodeling tumor suppressor genes, specifically the switch/sucrose non-fermenting (SWI/SNF) complex subunit genes *Arid1b*, *Arid1a*, and *Pbrm1* (Aiderus et al., 2021). This co-occurrence suggests that stabilized, truncated ZMIZ1 acts as an oncogenic driver that upregulates tumor susceptibility gene expression and may need concurrent epigenetic disruption in other factors to progress a benign lesion to malignant skin cancer.

### 6.2 Control of ZMIZ1 availability and regulation of AR-signaling is involved in prostate cancer

Prostate cancer (PCa) is a type of adenocarcinoma that develops from the gland cells in the prostate. Its high prevalence and morbidity are significant, ranking as the fifth leading cause of cancer-related death in men worldwide (Kania et al., 2025). While those with early-stage prostate cancer have a higher chance of survival, PCa can often go undiagnosed, progressing to castration-resistant prostate cancer (CRPC), which currently has no cure (Semenas et al., 2012; Saad et al., 2015). Therefore, research into novel diagnostic biomarkers and treatments for advanced stages is crucial.

Genome-wide association studies in both the Japanese and Chinese populations identified *ZMIZ1* as a susceptibility locus for PCa (Zhang et al., 2022b; Takata et al., 2019). SNPs rs777911174, rs4554825, and rs704017 were found to be independently associated with increased risk of PCa by altering the expression of ZMIZ1 (Takata et al., 2019; Daniunaite et al., 2021). These studies suggested the importance of tight regulation of ZMIZ1 expression. Subsequent genomic and functional studies have further demonstrated that multiple mechanisms that may affect ZMIZ1 availability, control of transcription, and activity of the AR-signaling pathway, which is strongly associated with prostate carcinogenesis.

Evidence that epigenetic control is a potential mechanism underlying *ZMIZ1* expression dysregulation in PCa was provided by Daniunaite and colleagues (Daniunaite et al., 2021). This study characterized methylome data from cancerous prostate tissue, noncancerous prostate tissue (NPT), and benign prostatic hyperplasia (BPH) samples (Daniunaite et al., 2021). This study found a significantly higher methylation frequency for *ZMIZ1* in PCa samples compared to NPT and BPH samples, and consistently, ZMIZ1 expression was also lower in cancerous tissues compared to NPT. Notably, PCa patients exhibiting aberrant *ZMIZ1* promoter methylation had reduced biochemical recurrence (BCR)-free survival rates (Daniunaite et al., 2021). Given that atypical promoter methylation is an early event in carcinogenesis, abnormal *ZMIZ1* methylation could have clinical value as a novel prognostic biomarker.

While epigenetic silencing at the promoter lowers *ZMIZ1* transcript levels, back-splicing of exons 2–4 of the *ZMIZ1* gene produces circZMIZ1, a circular noncoding RNA isoform that is associated with tumorigenesis in PCa. Jiang and colleagues found that circZMIZ1 is more stable than the linear *ZMIZ1* RNA and was markedly upregulated in PCa cells (Jiang et al., 2020). Knockdown of circZMIZ1 significantly impaired PCa cell proliferation by increasing G1 population while decreasing cells in S-phase, indicating G1 cell-cycle arrest (Jiang et al., 2020). Although the tumorigenic activity of circZMIZ1 may be completely independent of ZMIZ1 function in PCa, it is interesting to point out that circRNAs can regulate the splicing and transcription of the genes that host them, in this case *ZMIZ1*, and that they can serve as templates for short peptides whose functions can be similar to their full-protein counterparts (Xiao et al., 2022; Ren et al., 2020). Given this context, it is interesting that circZMIZ1 directly influences androgen receptor (AR) signaling pathways, as is the case for ZMIZ1. Androgen signaling plays a crucial role in prostate gland function; however, hyperactive AR can promote PCa development (Balk, 2014). Treatment of PCa cell line with dihydrotestosterone (DHT) significantly upregulated circZMIZ1, while silencing of AR significantly suppressed circZMIZ1. In turn, silencing circZMIZ1 downregulates AR splice variant-7 (AR-V7), which is a key driver of CRPC progression (Jiang et al., 2020). These results do not provide evidence that circZMIZ1 interacts with ZMIZ1, but they may suggest a possible convergence that would need to be further explored. These results also indicate that circZMIZ1 may act as an oncogene in PCa cell proliferation. This observation could prove useful as a novel diagnostic serum biomarker and a therapeutic target.

Beyond the circZMIZ1 interaction with AR signaling, the full-length ZMIZ1 protein directly modulates transcriptional activity of AR signaling. AR has an N-terminal transactivation domain which contains the polyglutamine (polyQ) tract. The length of the polyQ tract is inversely correlated with prostate cancer risk; specifically, shorter sequences (<20 Qs) increase risk, whereas longer sequences appear to offer a protective effect (Li et al., 2011; He et al., 2020). Given that ZMIZ1 is an AR coactivator, Li and colleagues studied its role in regulating the activities of AR with different lengths of polyQ tracts in prostate cancer (Ghashghaei et al., 2019). They found that ZMIZ1 directly binds to the N-terminal domain and enhances activity of AR with shorter polyQ lengths in the presence of physiological levels of androgens in PCa cell lines. ZMIZ1 had stronger protein-protein interaction with shorter polyQ tracts. In addition, cells co-expressing a shorter poly-Q tract with ZMIZ1 were found to have significant resistance to inhibition by anti-androgens, suggesting ZMIZ1-mediated amplification of shorter polyQ tracts facilitating escape from anti-androgen blockade in PCa cells. Mechanistically, the components of SWI/SNF complexes such as Brg1 and BAF57 were shown to be involved in the enhancement of ZMIZ1 on AR activity (Ghashghaei et al., 2019).

Current treatments for PCa include radiation (XRT) and androgen deprivation therapy (ADT), however, many patients develop resistance or recurrence (Kupelian et al., 2006). Given ZMIZ1’s pivotal involvement in AR co-activation and anti-androgen resistance, its expression is likely to directly impact clinical responses to combination therapies. Enzalutamide (ENZA), a novel androgen receptor antagonist, has recently shown promise in enhancing the effects of radiation therapy (Ghashghaei et al., 2019). Gene expression profiling of ENZA-induced radiosensitive signatures in PCa cells showed that combination treatments ENZA + XRT and ENZA + XRT + ADT significantly reduced ZMIZ1 expression compared to XRT-treatment alone (Ghashghaei et al., 2019). This suppression suggests that downregulating ZMIZ1 mediates the enhanced effects of radiation by ENZA, highlighting ZMIZ1 as a key therapeutic target to overcome treatment resistance.

### 6.3 ZMIZ1 amplifies ER signaling in breast cancer

Breast cancer (BC) is the most common form of cancer in women worldwide (Łukasiewicz et al., 2021). 75% of breast cancers are Estrogen Receptor positive (ER+), meaning that the cancer cells contain estrogen receptors that are required for the cancer to grow and proliferate (Zhao et al., 2024). In ER + breast cancer, the receptors are no longer correctly regulated, leading to increased transcriptional activity favoring tumorigenesis. Zhao and colleagues identified ZMIZ1 as an estrogen receptor coactivator that promotes the growth of ER + breast cancer (Zhao et al., 2024). Clinical and transcriptomic analyses of patient tumor samples showed that *ZMIZ1* co-expresses with ER and exhibits higher activity in ER + breast cancer. Interestingly, higher expression of ZMIZ1 correlates with lower overall survival of ER + patients, highlighting its potential role in tumor progression and as a marker of aggressiveness. Mechanistically, functional studies in breast cancer cell lines demonstrated that ZMIZ1 acts as part of the ER-α transcriptional complex by binding to ER, which in turn co-binds the promoter of the cell-cycle regulator E2F2. This interaction enhances the transcriptional activation of E2F2 and a subset of ER-regulated cell-cycle target genes (Zhao et al., 2024). Conversely, ZMIZ1 knockdown significantly reduces ER transcriptional activity, delays the estrogenic expression of E2F2, and suppresses ER + breast cancer cell proliferation, possibly by impairing cell cycle progression.

Evidence supporting an indirect association between *ZMIZ1* and triple-negative breast cancer (TNBC) was suggested by the discovery of rs704010 (Shan et al., 2019). The triple-negative breast cancer (TNBC) subtype accounts for 15%–20% of breast cancer and has a higher rate of early relapse and mortality (Shan et al., 2019). TNBC does not contain any of the receptors found in breast cancer, including the estrogen receptor. The lack of targetable receptors makes it difficult to treat, leading it to have one of the poorest prognoses among all breast cancer subtypes. Recently, chemokine ligand 5 (CCL5) has been found to play a major role in recruiting immune cells into the tumors, and treatment to increase CCL5 has been associated with increased responsiveness to immunotherapy and increased survival (Shan et al., 2019). Because ZMIZ1 is important for T-cell development and could regulate CCL5 expression via NOTCH signaling, this variant could affect the level of CCL5 and responsiveness of the tumor. Overall, the role of ZMIZ1 as an ER coactivator is supported; however, the potential of the *ZMIZ1* SNP in predicting BC risk and disease aggressiveness would require further investigation.

### 6.4 Genetic association and post-translational control of SIRT1/FOXO3a axis by *ZMIZ1* in colorectal cancer

*ZMIZ1* has been implicated in cancers of the digestive system, including colorectal cancer (CRC). The association between ZMIZ1 expression and CRC is complex. Data from The Cancer Genome Atlas (TCGA) (The Cancer Ge nome Atlas Network, 2012) and the Catalogue of Somatic Mutation in Cancer (COSMIC) (Forbes et al., 2011) indicate *ZMIZ1* gene expression is reduced in colon tumor tissue compared with normal colon tissue.

Evidence from genetic association studies across diverse populations consistently identified risk locus rs704017, which is in intron three of long non-coding ZMIZ1 Antisense RNA 1 (*ZMIZ1-AS1)*, as a key susceptibility locus for CRC (Genetics and Epidemiology of Colorectal Cancer Consortium GECCO et al., 2014; Song et al., 2017). It has been proposed that *ZMIZ1-AS1* interferes with and inhibits ZMIZ1 translation (Song et al., 2017). Specifically, GWAS studies in East Asian cohorts showed that rs704017 confers an age-dependent risk, showing a higher allele frequency in late-onset CRC (>50 years) relative to early-onset cases (Genetics and Epidemiology of Colorectal Cancer Consortium GECCO et al., 2014; Song et al., 2017). Additionally, candidate SNP genotyping in European populations further linked rs704017 to increased susceptibility to serrated polyposis syndrome, a pre-malignant condition that can lead to CRC (Arnau-Collell et al., 2020). These studies do not report how this SNP may affect the level of expression of *ZMIZ1-AS1* or *ZMIZ1.* Further investigation is needed to understand how rs704017 may increase risk of CRC.

Functional studies showed that knockdown of ZMIZ1 in colon cancer cell lines inhibited cell proliferation and autophagy while inducing apoptosis (Huang et al., 2024). A potential mechanism by which ZMIZ1 may control fate of colon cancer cells is by altering the SIRT1/FOXO3a axis. ZMIZ1 mediates ubiquitin-dependent degradation of SIRT1 via its E3 ubiquitin ligase activity. ZMIZ1 knockdown in human colon cancer cell lines decreased Ubiquinated-SIRT1, which resulted in increased levels of SIRT1 and reduced acetylated-FOXO3a (Huang et al., 2024). SIRT1 is a NAD-dependent histone/protein deacetylase that plays important functions in cellular homeostasis and has a complex role in carcinogenesis of colon cancer. A specific substrate of SIRT1 is FOXO3a. SIRT1 regulates the transcriptional activity of FOXO3a on target genes by deacetylating FOXO3a, which is a key balancer linking autophagy and apoptosis through transcriptional regulation (Huang et al., 2024). Overall, this study showed that ZMIZ1 knockdown significantly inhibited proliferation and autophagy and induced apoptosis in cancer cells. These findings suggest that ZMIZ1 might have a pro-cancer role in promoting the malignant proliferation of colon cancer through regulating the SIRT1/FOXO3a axis and might be a potential therapeutic target.

### 6.5 *ZMIZ1-AS1* promotes osteosarcoma progression via PTBP1-Dependent *ZMIZ1* mRNA stabilization in osteosarcoma

Osteosarcoma (OS) is the most common cancer of the bone and is associated with a high risk of metastasis and recurrence. The long non-coding RNA (lncRNA) have been found to affect OS oncogenesis by regulating their corresponding sense transcripts, including *ZMIZ1-AS1*. In OS tissues and cell lines, inactivation of the Hippo signaling pathway allows the transcription factors SOX2 and MYC to directly bind to the promoter of lncRNA *ZMIZ1-AS1*, upregulating its expression. Once transcribed, *ZMIZ1-AS1* acts as a molecular scaffold that recruits the RNA-binding protein PTBP1 to bind and stabilize *ZMIZ1* mRNA. This higher expression of ZMIZ1 protein drives aggressive malignant phenotypes, whereas silencing *ZMIZ1-AS1* represses OS cell viability, proliferation, migration, and invasion both *in vitro* and *in vivo* (Zhou Y. et al., 2022) Similarly, other lncRNAs such as DNAJC3-AS1, ZFAS1, FEZF1-AS1, and ZEB2-AS1 have been identified to promote osteosarcoma development and progression through various mechanisms, such as regulating DNAJC3, SRSF3, miR-4443/NUPR1 axis, and ZEB2, respectively (Yang et al., 2023; Zhou et al., 2018; Zhang Y. et al., 2022; Liang et al., 2019). These findings suggest a mechanisms of stabilization of ZMIZ1 is pro-oncogenic in OS, and highlight the significant impact of long non-coding RNAs on osteosarcoma pathogenesis and suggest their potential as prognostic biomarkers and therapeutic targets in the treatment of this aggressive bone cancer.

### 6.6 Intragenic DNA methylation of *ZMIZ1* in glioblastoma

Glioblastoma is the most common brain tumor and is characterized by its malignant nature, aggressiveness and resistance to therapy. Aberrant DNA methylation has been established as a tumor-promoting factor, particularly at promoter regions (Nishiyama and Nakanishi, 2021). Mathios and colleagues investigated the role of DNA methylation in intragenic regions of ZMIZ1 in the context of glioblastoma tumors (Mathios et al., 2019). This study found an intragenic region in ZMIZ1, where hypomethylation was associated with lower patient survival. At this region, hypomethylation leads to higher expression of an alternative transcript encoding a truncated *ZMIZ1* transcript that is more stable and undergoes proteasomal degradation at a slower rate than full-length ZMIZ1. This hyperstable ZMIZ1 isoform was positively associated with aggressive glioblastoma phenotype leading to poor patient survival.

Functional studies validated these clinical observations; *In vitro* knockdown of *ZMIZ1* in human gliobastoma cell lines showed a decrease in migration, while overexpression of both the full and truncated transcript increased the migratory potential. Similarly, *in vivo* study in mice showed decreased rates of tumor growth upon *Zmiz1* knockdown (Mathios et al., 2019). Consistently with some other cancerous cell contexts, these studies support that increased stability of ZMIZ1 transcript may have a pro-oncogenic effect and underscore the importance of intragenic DNA methylation as a promising molecular marker with implications for cancer prognosis and treatment strategies.

### 6.7 ZMIZ1 associations in renal and hepatic malignancies

Renal Cell Carcinoma (RCC) originates from the tubular epithelial cells of the kidney and represents the third most common cancer of the urinary system, accounting for over 131,000 deaths annually (Safiri et al., 2020). A meta-analysis of ten Gene Expression Omnibus (GEO) datasets identified *ZMIZ1* to be downregulated in RCC, potentially driving the suppression of normal renal metabolic pathways (Ioannou et al., 2023). Additionally, using Connectivity Map (CMap) screening, the study identified putative repurposed small-molecule drugs such as Etoposide that may be capable of reversing this *ZMIZ1*-associated downregulated gene signature to inhibit kidney cancer cell growth *in vitro* (Ioannou et al., 2023).

Primary Intrahepatic Cholangiocarcinoma is the second most common liver malignancy originating from the epithelial lining of the bile ducts inside the liver. Information linking ZMIZ1 to liver cancer is scarce, and the available findings appear paradoxical regarding the potential direction of the association. Using a dataset of patients with Intrahepatic Cholangiocarcinoma (ICC), a study found that high ZMIZ1 expression correlated with better prognosis suggesting a potential use as a protective biomarker. However, functional experiments in mice showed that ZMIZ1 acts as a core transcription factor required for liver progenitors or epithelial cells to transform into ICC lesions, and *in-vivo* knockout of *Zmiz1* led to the blockade of ICC initiation and progression, suggesting a pro-oncogenic effect (Wang et al., 2022). These apparently opposing observations may reflect differences between ZMIZ1 expression as a prognostic marker and its functional role in tumor initiation and maintenance. More studies are needed to understand the potential association of ZMIZ1 in this context.

## 7 Context-dependent and potentially opposing roles of ZMIZ1

A rigorous synthesis must account for the possibility that ZMIZ1 exerts context-dependent, and potentially opposing, effects and that the direction of reported associations may vary across biological settings. Rather than undermining the case for ZMIZ1 as a central regulator, these apparent contradictions are predicted by the partner- and dosage-dependent framework outlined above. The clearest example is in oncology, where increased ZMIZ1 expression and/or stability promotes cancer proliferation in some tissues; while in other tissues, the reduction of ZMIZ1 is associated with cancer or worse outcomes. Overexpression of ZMIZ1, or of the truncated Zmiz1Δ185 isoform, drives cutaneous squamous cell, prostate, and estrogen-receptor-positive breast carcinomas, and *ZMIZ1* knockdown suppresses tumor growth in these models. However, the expression of ZMIZ1 is reduced in colorectal tumor tissue and renal cell carcinoma compared to normal tissues. Although the association between reduced ZMIZ1 expression and cancer in these contexts does not imply a protective role of ZMIZ1, it illustrates that both increased and decreased levels of ZMIZ1 are associated with cancer in different tissues. Intrahepatic cholangiocarcinoma illustrates the tension within a single tumor type, where high *ZMIZ1* expression predicts better prognosis yet ZMIZ1 is required as a core transcription factor for tumor initiation.

Directional inconsistencies extend beyond cancer. In multiple sclerosis, rs1250550 has been reported as a risk variant for MS, and low *ZMIZ1* expression confers risk, the opposite polarity to the gain of expression seen in epithelial cancers. In inflammatory bowel disease, ZMIZ1 is predominantly a risk locus, yet the rs1250550 variant was associated with a decreased risk of Crohn’s disease in a pediatric cohort. Most strikingly, in Lewy body disease, the ZMIZ1 rs6480922 variant is protective, associating with reduced neuropathological burden–an effect direction opposite to its risk association in most other conditions. We interpret these divergences as reflecting cell-type-specific partner availability and tissue-specific dosage sensitivity rather than as irreconcilable discrepancies; nonetheless, several rest on single cohorts or on locus-level associations that have not been fine-mapped to ZMIZ1 itself (some implicate the antisense transcript *ZMIZ1*-AS1) and should be regarded as provisional. Table 1 summarizes the principal ZMIZ1–disease relationships, the system studied, the class and tier of supporting evidence, and the direction of the ZMIZ1 change, making the strength and polarity of each claim explicit.

## 8 Clinical implications and therapeutic potential: current status and limitations

The range of conditions in which ZMIZ1 has been implicated has naturally raised interest in its potential utility as a diagnostic or prognostic biomarker. Therefore, it is important to clearly define the current state of the evidence and distinguish from promising applications that remain to be validated.

Pathogenic ZMIZ1 variants are now reported by clinical gene panels for hereditary developmental disorders, including panels covering intellectual disability, autism and epilepsy. For families who may have spent years seeking an explanation for their child’s developmental delay, identification of a pathogenic *ZMIZ1* variant can establish a molecular diagnosis, allows recurrence risk to be estimated, and connects them to others with the same diagnosis. However, the current clinical utility remains primarily diagnostic: management remains supportive and symptom-directed, and no ZMIZ1-specific intervention is currently available. As further variants are reported and genotype-phenotype correlations sharpen, the prognostic information a diagnosis carries should improve.

ZMIZ1 potential use in prognosis and stratification still remains at the stage of retrospective correlation. Elevated ZMIZ1 expression marks particular molecular subtypes of T-cell leukemia, and ZMIZ1 expression and activity correlate with outcome in prostate and ER-positive breast cancer cohorts. These correlations have not been tested prospectively, no assay has been analytically validated, and no threshold has been defined at which a clinician would act differently. Therefore, currently evidence remains hypothesis-generating rather than sufficient to support its use as prognosis or stratification biomarker.

The suggestion of the potential use of circulating tumor DNA might be used to follow ZMIZ1 faces a more basic obstacle: such assays primarily detect recurrent sequence alterations, whereas the ZMIZ1 changes described in this review are predominantly changes in expression, methylation, isoform usage and transcript stability. Thus, outside the tumor-specific genetic alterations discussed below, the potential of this strategy remains hypothetical.

Direct therapeutic targeting is, for now, a proposal rather than a program. The appeal is clear enough: an agent that blocked a single ZMIZ1 interface, for example, blocking the NOTCH1 interaction, or the surface engaged by nuclear receptors, might suppress one ZMIZ1-dependent program while leaving other functional components intact. This is something lowering ZMIZ1 levels will not achieve. However, no such agent has been reported yet. Peptides designed to occlude protein-protein interfaces face well-recognized problems of stability and intracellular delivery. RNA interference has been used to deplete ZMIZ1 in cancer cell lines and mouse models, where it slows tumor growth, but these still remain as laboratory tools rather than therapeutic agents. The one exception is the ZMIZ1-ABL1 fusion, which retains the ABL1 kinase domain and is on that basis expected to respond to tyrosine kinase inhibitors. However, the reported cases are too few to indicate how well, or for how long these inhibitors may work. Currently, no ZMIZ1-directed agent has entered clinical development.

Attempts to drug ZMIZ1 will encounter the same dosage sensitivity that makes it biologically interesting. In humans, heterozygous loss of function causes a syndromic neurodevelopmental disorder, thus, approximately halving ZMIZ1 is already not tolerated in the developing nervous system. Conditional deletion in mice disrupts blood-vascular and lymphatic development, T-cell development, and cortical projection-neuron specification. In pancreatic β-cells, loss and gain are both detrimental, which means the dose–response relationship is not monotonic, and a partial inhibitor cannot be assumed to be proportionately safer than a complete one. A systemically delivered ZMIZ1 inhibitor should therefore be expected to have a narrow therapeutic index, with the anticipated liabilities concentrated in vascular and lymphatic homeostasis, lymphopoiesis and glucose regulation; on current evidence, exposure during development would be contraindicated.

Two features of ZMIZ1 biology suggest where the standard for clinical application might be met. The first is the modular architecture described above: because different partners engage separate domains (Figure 2), selectivity is at least structurally possible, and blocking one interface is a more defensible goal than reducing the protein. Whether it can be realized depends on structural characterization of individual ZMIZ1-partner complexes, which makes that work a prerequisite. The second is that several of the pro-tumorigenic species are absent from normal tissue: the Zmiz1-185 truncation, the hyperstable isoform arising from intragenic hypomethylation in glioblastoma, circZMIZ1, and the ZMIZ1-ABL1 fusion. Directing an agent at a species that healthy cells do not express would avoid much of the toxicity problem outlined above, though each presents its own difficulty: a truncated protein and a circular RNA are not equally tractable, and none has been targeted experimentally. We regard these tumor-restricted species, together with interface-selective blockers, as the more promising of the available routes rather than expression-level intervention. Both routes remain to be attempted.

## 9 Conclusion and future directions

ZMIZ1 emerges as a multifunctional partner-dependent transcription regulator with diverse roles across biological systems and significant implications for human health and disease. Its complex interactions with transcription factors, signaling pathways, and chromatin regulators enable it to orchestrate gene expression programs controlling development, differentiation, and homeostasis in multiple tissues. Disruption of these finely tuned regulatory networks through mutation, aberrant expression, or altered function of ZMIZ1 contributes to various pathological conditions ranging from neurodevelopmental disorders to cancer. The partner-directed rheostat model advanced here is a working hypothesis. It accounts economically for the breadth of ZMIZ1 phenotypes and for the fact that both loss and gain of function are pathological, but it has not been tested directly in many of the contexts described. Alternative mechanisms, including partner-independent activities of ZMIZ1 and locus-level effects that do not act through ZMIZ1 protein at all, are also possible. This working model will evolve over time as new evidence becomes available and clarify ZMIZ interactions and functions.

The molecular versatility of ZMIZ1 is reflected in its ability to modulate multiple signaling pathways, including Notch and nuclear receptors, each with context-specific outcomes. This functional diversity arises from ZMIZ1’s modular protein structure, which facilitates interactions with numerous partner proteins and enables precise coordination of transcriptional responses. As research continues to unravel the complex biology of ZMIZ1, new opportunities for diagnostic and therapeutic applications will likely emerge.

ZMIZ1is implicated as a susceptibility gene in autism, ID, type 2 diabetes, autoimmune disorders, and cancer (Figure 3). However, the precise molecular and signaling mechanisms underlying its involvement in these diseases remain incompletely understood, necessitating further research to potentially develop new therapeutic strategies (Figure 3). *ZMIZ1* exhibits dynamic expressions during embryogenesis, particularly in vascular development, brain tissues, and gonads, suggesting multifaceted roles in physiological functions. Could its role in gonad development be related to the progression of sex-related prostate, ovarian, and breast cancer? Does ZMIZ1’s role in neurogenesis and synaptic signaling lend itself to intellectual disability and autism when aberrantly expressed? Exploring its specific contributions to developmental processes could elucidate pathways linking ZMIZ1 dysregulation to disease pathogenesis, including potential roles in autism, intellectual disability, autoimmune disorders, and cancers.

Future directions in ZMIZ1 research should address several key areas. First, more comprehensive mapping of ZMIZ1’s tissue-specific interactomes would clarify how the same protein can mediate distinct functions across different cellular contexts. Second, structural studies of ZMIZ1 in complexes with its various binding partners would facilitate rational drug design for therapeutic targeting. Third, investigating signaling pathways regulated by ZMIZ1 and exploring inter-pathway crosstalk would help us understand how it is involved in multiple diseases. It would be worthwhile to determine how reported mutations of *ZMIZ1* impact its structure and function in transcription and overall signaling. Fourth, improved animal models with conditional and inducible manipulation of ZMIZ1 would enhance our understanding of its temporal and spatial requirements in development and disease. Finally, clinical correlation studies linking ZMIZ1 status with treatment responses would help identify patient populations most likely to benefit from ZMIZ1-directed interventions.

As our understanding of ZMIZ1 biology continues to expand, this versatile transcription cofactor promises to yield new insights into fundamental biological processes and innovative approaches to addressing human disease.
